# Revision of the Late Jurassic teleosaurid genus *Machimosaurus* (Crocodylomorpha, Thalattosuchia)

**DOI:** 10.1098/rsos.140222

**Published:** 2014-10-15

**Authors:** Mark T. Young, Stéphane Hua, Lorna Steel, Davide Foffa, Stephen L. Brusatte, Silvan Thüring, Octávio Mateus, José Ignacio Ruiz-Omeñaca, Philipe Havlik, Yves Lepage, Marco Brandalise De Andrade

**Affiliations:** 1 School of GeoSciences, University of Edinburgh, The King's Buildings, Edinburgh EH9 3JW, UK; 2 School of Ocean and Earth Science, National Oceanography Centre, University of Southampton, Southampton SO14 3ZH, UK; 3 Le Musée des Dinosaures d'Espéraza, Espéraza 11260, France; 4 Department of Earth Sciences, Natural History Museum, London SW7 5BD, UK; 5 School of Earth Sciences, Wills Memorial Building, University of Bristol, Bristol BS8 1RJ, UK; 6 National Museums Scotland, Chambers Street, Edinburgh, EH1 1JF, UK; 7 Naturmuseum Solothurn, Klosterplatz 2, Solothurn 4500, Switzerland; 8 Departamento de Ciências da Terra, Faculdade de Ciências e Tecnologia, Universidade Nova de Lisboa, GeoBioTec, Quinta da Torre, Caparica 2829-516, Portugal; 9 Museu da Lourinhã, Rua João Luis de Moura 9, Lourinhã 2530-158, Portugal; 10 Museo del Jurásico de Asturias (MUJA), Colunga 33328, Spain; 11 Paläontologische Sammlung, Senckenberg Center for Human Evolution and Palaeoenvironment, Eberhard Karls Universität, Sigwartstrasse 10, Tübingen 72072, Germany; 12 Sciences et Géologie Normandes, Le Havre 76620, France; 13 Faculdade de Biociências, Pontifícia Universidade Católica do Rio Grande do Sul, Avenida Ipiranga 6681, Porto Alegre 90619-900, Brazil

**Keywords:** Africa, Europe, Kimmeridgian, *Machimosaurus*, Teleosauridae, Tithonian

## Abstract

*Machimosaurus* was a large-bodied genus of teleosaurid crocodylomorph, considered to have been durophagous/chelonivorous, and which frequented coastal marine/estuarine ecosystems during the Late Jurassic. Here, we revise the genus based on previously described specimens and revise the species within this genus. We conclude that there were three European *Machimosaurus* species and another taxon in Ethiopia. This conclusion is based on numerous lines of evidence: craniomandibular, dental and postcranial morphologies; differences in estimated total body length; geological age; geographical distribution; and hypothetical lifestyle. We re-diagnose the type species *Machimosaurus hugii* and limit referred specimens to only those from Upper Kimmeridgian–Lower Tithonian of Switzerland, Portugal and Spain. We also re-diagnose *Machimosaurus mosae*, demonstrate that it is an available name and restrict the species to the uppermost Kimmeridgian–lowermost Tithonian of northeastern France. We re-diagnose and validate the species *Machimosaurus nowackianus* from Harrar, Ethiopia. Finally, we establish a new species, *Machimosaurus buffetauti*, for the Lower Kimmeridgian specimens of France and Germany (and possibly England and Poland). We hypothesize that *Machimosaurus* may have been analogous to the Pliocene–Holocene genus *Crocodylus* in having one large-bodied taxon suited to traversing marine barriers and additional, geographically limited taxa across its range.

## 2. Introduction

Teleosaurids were a successful and diverse group of marine crocodylomorphs that lived during the Jurassic. Most teleosaurids are often considered to be marine analogues to extant gavials, due to their elongate, tubular, polydont snout, presumed primarily piscivorous diet and dorsally directed orbits [1–5]. However, there is great confusion surrounding the taxonomy of one of the most characteristic teleosaurid genera: *Machimosaurus*. This genus is often considered to be durophagous/chelonivorous due to a suite of craniodental morphologies that would have been well suited for feeding on hard-shelled turtles or thick-scaled fish: i.e. a foreshortened snout, proportionally enlarged supratemporal fenestrae and blunt, heavily ornamented dentition [3,5–8]. As such, it is one of the more unusual crocodylomorphs of the Jurassic.

Two recent papers have hypothesized some unusual subjective species synonymies for the type species *Machimosaurus hugii* and made confusing statements about the type specimen of this species [9,10], while one has questioned the availability of a second species, *Machimosaurus mosae*, as a taxonomic name [10]. This is the impetus for this study. Here, we undertake a systematic revision of *Machimosaurus* and demonstrate that there were three species in the Kimmeridgian–Tithonian of Europe and a fourth species in Ethiopia. The third European species is a new taxon we name herein for the Lower Kimmeridgian specimens from France and Germany. The three European species were non-sympatric and differed in craniomandibular, dental and postcranial morphologies, total body length, geological age, geographical distribution and hypothetical lifestyle. We also address the issues surrounding the type specimens of these species and demonstrate that *M. mosae* is indeed an available name.

### 2.1 Institutional abbreviations

BHN2R, Muséum d'Histoire Naturelle de Boulogne-sur-Mer, France (closed in 2003); DFMMh, Dinosaurier-Freilichtmuseum Münchehagen, Lower Saxony, Germany; GPIT, Paläontologische Sammlung der Eberhard Karls Universität Tübingen, Germany; MCNV, Museo de Ciencias Naturales de Valencia, Spain; MG, Museu Geológico, Lisbon, Portugal; ML, Museu da Lourinhã, Portugal; MPV, Musée paléontologique (Paléospace) de Villers-sur-Mer, Normandy, France; MUJA, Museo del Jurásico de Asturias, Colunga, Spain; NHMUK, Natural History Museum, London, United Kingdom; NMS, Naturmuseum Solothurn, Switzerland; OUMNH, Oxford University Museum of Natural History, United Kingdom; RBINS, Royal Belgian Institute of Natural Sciences, Brussels, Belgium; SMNS, Staatliches Museum für Naturkunde Stuttgart, Germany; TWCMS, Sunderland Museum and Art Gallery, United Kingdom.

## 3. *Machimosaurus* through time

### 3.1 Bathonian

Based upon isolated tooth crowns from the Bathonian of France, Sauvage [11] established two species: *Machimosaurus bathonicus* and *Machimosaurus rigauxi*. Krebs [7, p. 48] considered these teeth more likely to be from *Steneosaurus*, as their apices are too pointed and the enamel surfaces too smooth to belong to *Machimosaurus*. As blunt apices and numerous apicobasal enamel ridges are apomorphies of *Machimosaurus* (see below), these tooth taxa cannot be referred to this genus.

**Figure 1.**
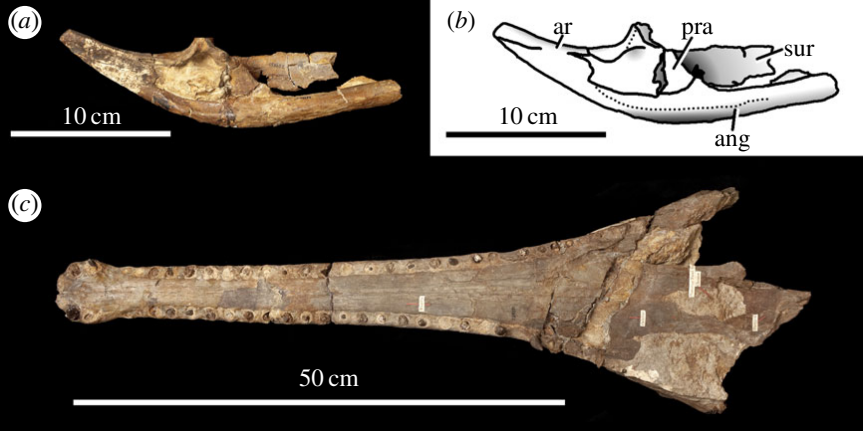
*Steneosaurus larteti*, OUMNH J.29850 and OUMNH J.29851, referred specimens. (*a*) Photograph and (*b*) line drawing of the posterior end of the left mandibular rami in medial view. (*c*) Photograph of the skull in palatal view. ang, angular; ar, articular; pra, prearticular; sur, surangular.

*Machimosaurus*/‘*Steneosaurus*’ *obtusidens*-like tooth crowns are also found in Bathonian deposits of the Great Oolite Group from England. One such tooth (TWCMS K1239) from Maidford in Northamptonshire has a blunt apex, and numerous apicobasal enamel ridges on the lingual and labial surface. This tooth crown is similar to the posterior-most tooth crowns of *Steneosaurus larteti* skulls (OUMNH J.29850 and OUMNH J.29851) from the Great Oolite Group of England (figure 1). It is therefore possible that the ‘*Machimosaurus*’ tooth taxa from France are also referable to *S. larteti*; however, investigating that hypothesis is beyond the scope of this study.

### 3.2 Callovian

‘*Steneosaurus*’*obtusidens* has been considered to be a subjective junior synonym of the Kimmeridgian taxon *M. hugii*, although most studies which drew this conclusion did note that further study was necessary on the anatomy and taxonomy of blunt-toothed teleosaurids [4,12,13]. ‘*Steneosaurus*’ *obtusidens* is known from the Oxford Clay Formation of central England, and a specimen from the Marnes de Dives Formation of northern France has been referred to this species [1,13,14]. Recent studies, however, have considered ‘*S*.’ *obtusidens* to be distinct enough to warrant its own genus [10,15], which we agree with. The holotype of this species is currently being re-described, which will help elucidate its anatomy and evolutionary relationships.

An isolated tooth crown (OUMNH J.14464) referred to *M. rigauxi* was found at Hanborough railway station, Oxfordshire, England (Cornbrash Formation) [16]. The description and figures of this specimen [16, p. 26–27, plate 1 fig. 5*a*–*c*] match the posterior dentition of the ‘*S*.’ *obtusidens* holotype, as the carinal keels are prominent and the apicobasal enamel ridges near the keels converge and contact the keel itself [1,15].

Based upon the Bathonian tooth taxa being more similar to *Steneosaurus* (in particular *S. larteti*) and ‘*S*.’ *obtusidens* being a distinct taxon, *Machimosaurus* is therefore unknown in the Middle Jurassic.

### 3.3 Oxfordian

**Figure 2.**
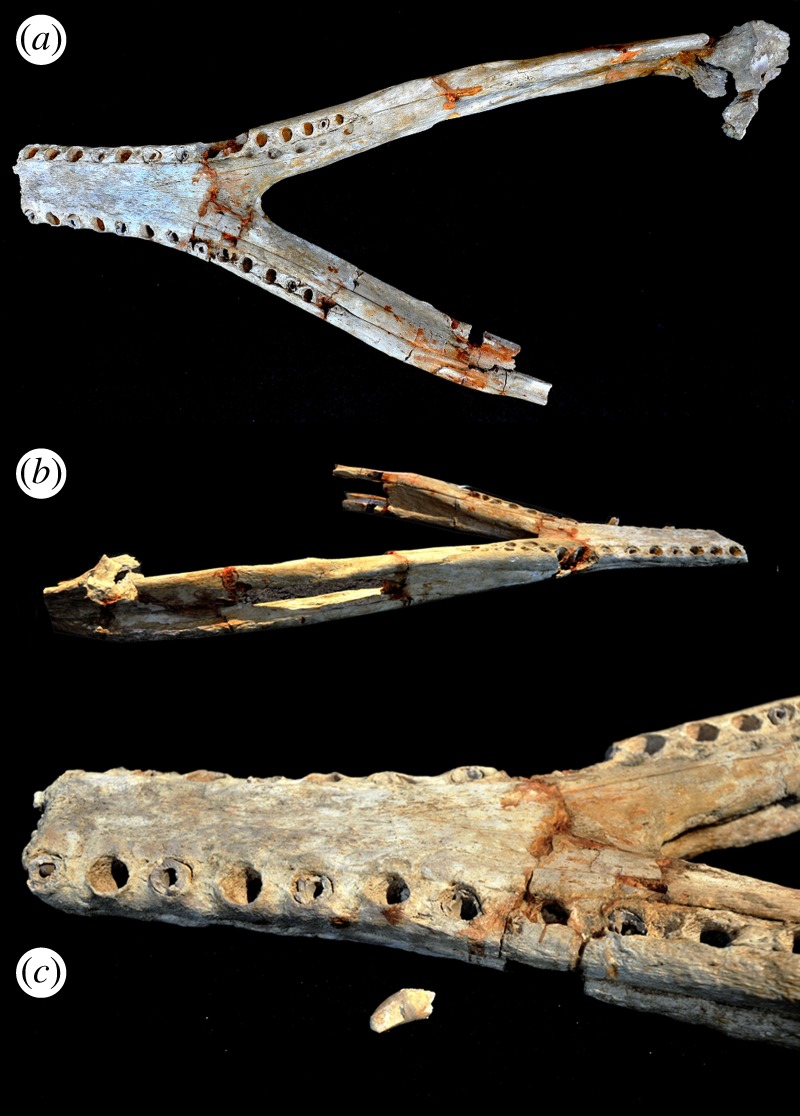
*Machimosaurus* sp., Musée de la Princerie (Verdun, France) 2007.0.14. Incomplete lower jaw in (*a*) dorsal view, (*b*) right oblique view and (*c*) left oblique view.

An almost complete *Machimosaurus* mandible with isolated tooth crowns is known from the Upper Oxfordian (*Perisphinctes* *cautisnigrae* NW European ammonite zone, *Pe*. *variocostatus* subzone) of Haudainville near Verdun (Département de la Meuse, northeastern France) (figure 2). This mandible has previously been assigned to *Steneosaurus* cf. *obtusidens* and *Machimosaurus* cf. *hugii* (see [12] and the references therein). However, here we consider this specimen as *Machimosaurus* sp.

Moreover, two isolated tooth crowns are known from the Upper Oxfordian Calcaire gréseux d'Hennequeville Formation. These teeth were found at Villerville, Département du Calvados, Basse-Normandie, France [17, p. 97–98, fig. 2].

Sauvage [18] reported and figured the first *M. hugii* specimens from Portugal. These included Oxfordian specimens, such as an isolated tooth from the Upper Oxfordian of Cesareda ‘Couches à *Cidaris chofatti*’ [18, plate 3 fig. 10] and a partial snout from Malhão, Algarve (one specimen label has: ‘Entre Amendoeira et Azinhal, Flanc nord de Malhão prés Estoy’) which is from the same horizon as *Perisphinctes effrenatus* [18, plate 3 fig. 9, and plate 5 figs 6 and 7].

Moreover, an isolated *Machimosaurus* sp. tooth crown (ML1208) was collected from Middle Oxfordian deposits at Cesaredas (39°N, 9°W) in central west Portugal.

### 3.4 Oxfordian–Kimmeridgian

**Figure 3.**
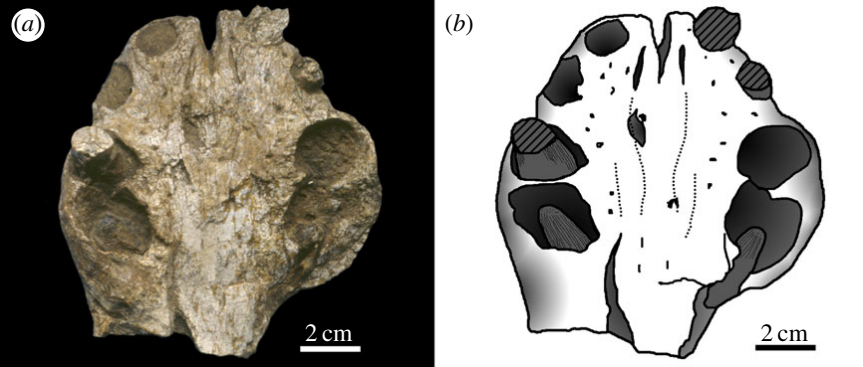
*Machimosaurus nowackianus* comb. nov., GPIT Orig. Huene 1938 fig. 1–4, holotype. Incomplete dentary in dorsal view, (*a*) photograph and (*b*) line drawing.

From near Harrar in Ethiopia (Oxfordian or Kimmeridgian aged deposits), an anterior region of dentary was described as a new species of pliosaurid sauropterygian, cf. *Simolestes nowackianus* [19] (figure 3). However, based on the dental morphology, the spatulate anterior region, arrangement of the dentary alveoli and thecodont tooth replacement, Bardet & Hua [20] demonstrated that it is in fact a large specimen of *Machimosaurus*. Currently, this is the only *Machimosaurus* specimen known from outside Europe.

### 3.5 Kimmeridgian

Prior to this study, two valid species of *Machimosaurus* were recognized in the Kimmeridgian of Europe: the type species *M. hugii* and *M. mosae* [4,21]. Based on the numerous European localities outlined below, during the Kimmeridgian *Machimosaurus* commonly frequented shallow marine ecosystems, with the occasional individual known from brackish and open-shelf environments, and possibly also freshwater environments.

**Figure 4.**
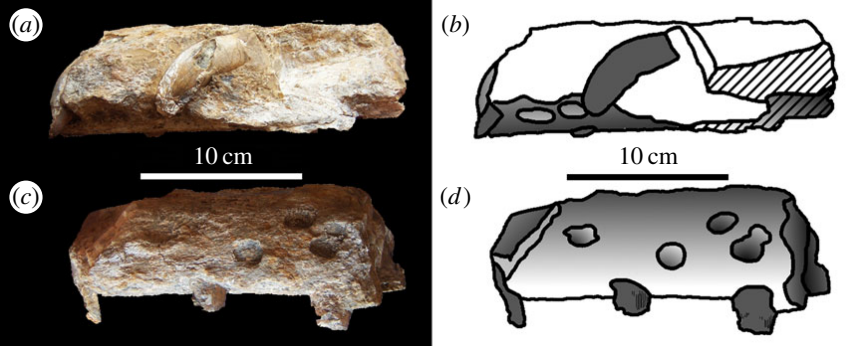
*Machimosaurus hugii*, MG-8730-1, referred specimen. Incomplete snout (fragment of maxilla), (*a*,*c*) photographs in both lateral views and (*b*,*d*) the corresponding line drawings in both lateral views.

**Figure 5.**
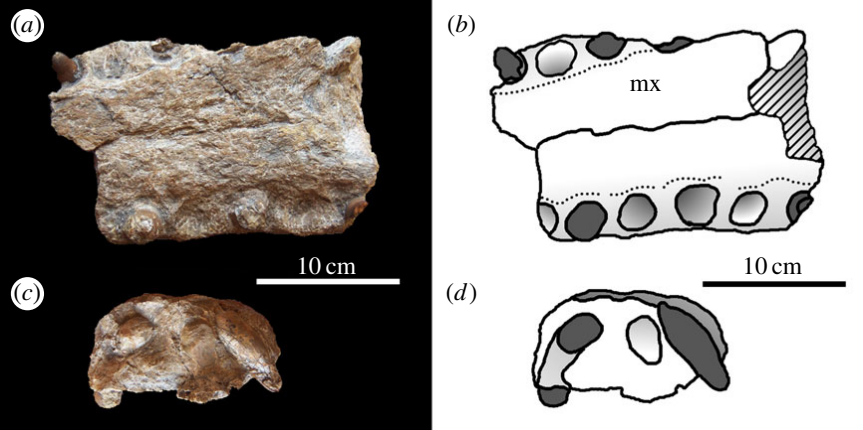
*Machimosaurus hugii*, MG-8730-1, referred specimen. Incomplete snout (fragment of maxilla), (*a*) photograph in ventral view, (*b*) line drawing in ventral view, (*c*) photograph in posterior view and (*d*) line drawing in posterior view. mx, maxilla.

**Figure 6.**
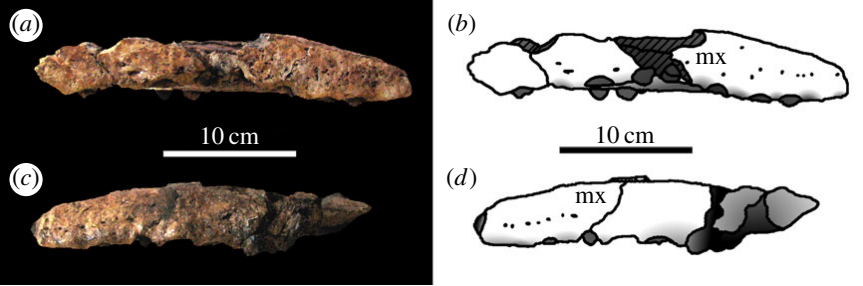
*Machimosaurus hugii*, MG-8730-1, referred specimen. Incomplete snout (fragment of maxilla, and possibly nasals), (*a*) photograph in right lateral view, (*b*) line drawing in right lateral view, (*c*) photograph in left lateral view and (*d*) line drawing in left lateral view. mx, maxilla.

**Figure 7.**
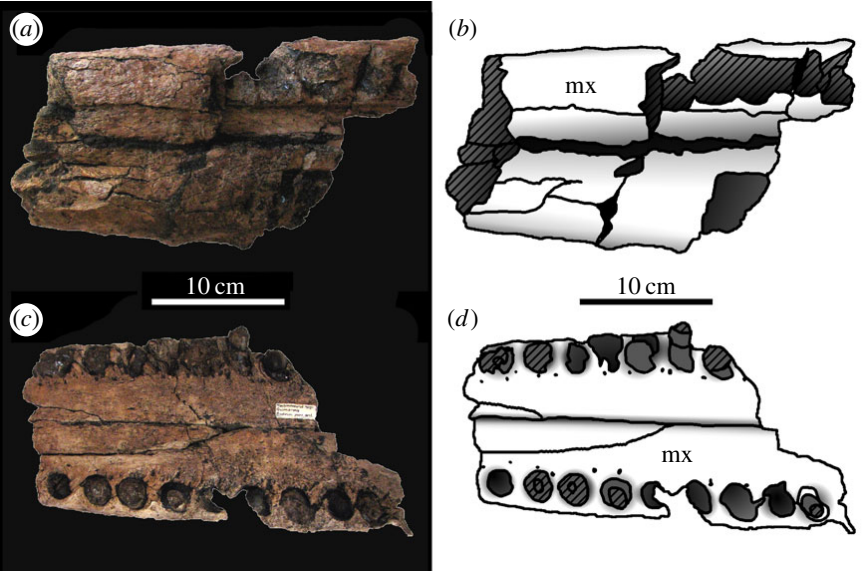
*Machimosaurus hugii*, MG-8730-1, referred specimen. Incomplete snout (fragment of maxilla, and possibly nasals), (*a*) photograph in dorsal view, (*b*) line drawing in dorsal view, (*c*) photograph in ventral view and (*d*) line drawing in ventral view. mx, maxilla.

**Figure 8.**
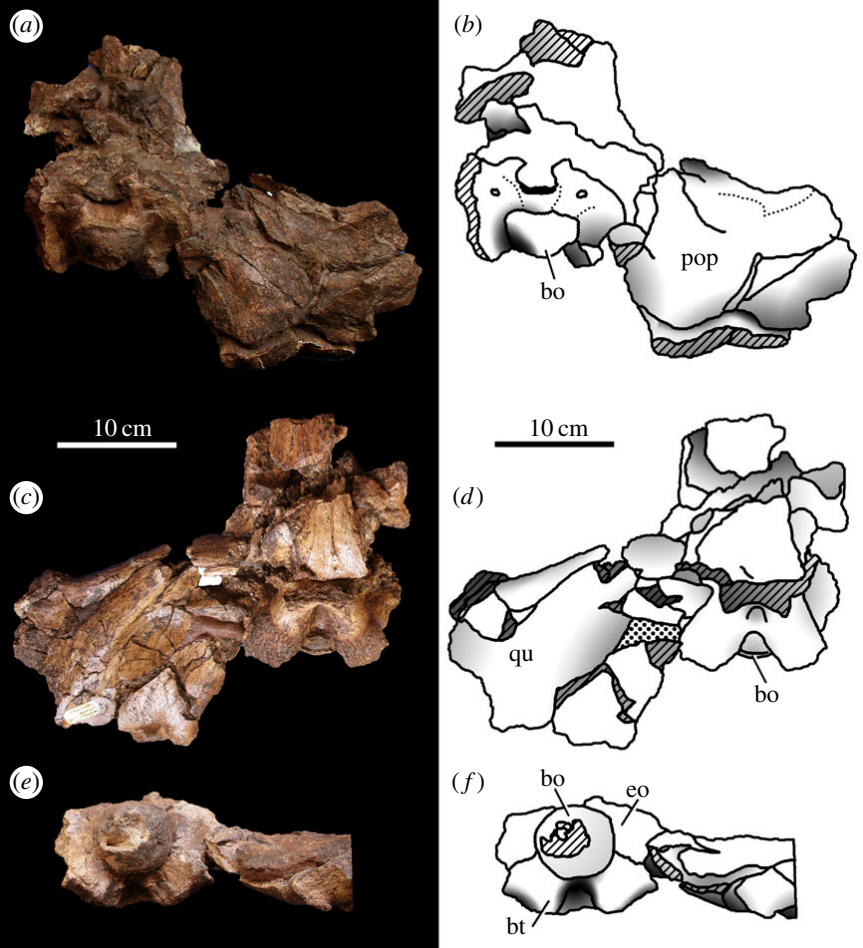
*Machimosaurus hugii*, MG-8730-2, referred specimen. Incomplete braincase, (*a*) photograph in dorsal view, (*b*) line drawing in dorsal view, (*c*) photograph in ventral view, (*d*) line drawing in ventral view, (*e*) photograph in occipital view and (*f*) line drawing in occipital view. bo, basioccipital; bt, basioccipital tuberosities; eo, exoccipital; pop, paroccipital process of the opisthotic; qu, quadrate.

**Figure 9.**
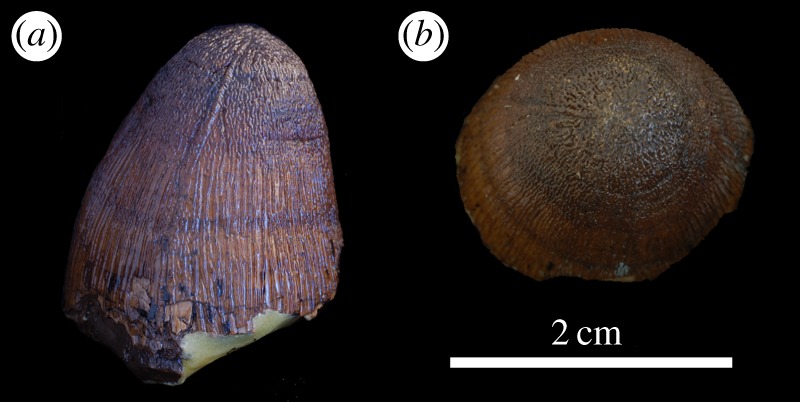
*Machimosaurus hugii*, MG unnumbered, referred specimen. Isolated tooth crown in (*a*) right lateral view and (*b*) apical view.

**Figure 10.**
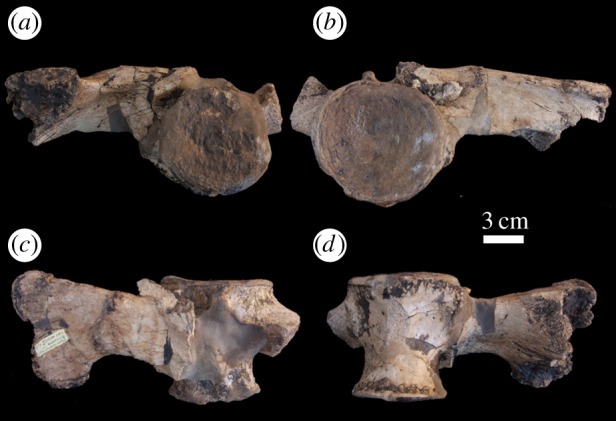
*Machimosaurus hugii*, MG unnumbered, referred specimen. First sacral vertebra in (*a*) posterior view, (*b*) anterior view, (*c*) dorsal view and (*d*) ventral view.

From Portugal, *Machimosaurus* is known from two sites:

— In 1943, the geologist Carlos Teixeira reported an isolated *M. hugii* tooth from Lagares (Colmeias, near Leiria), Portugal [22]. This tooth (MG 25) is from the Alcobaça Beds Formation (Upper Kimmeridgian).
— The largest known specimen of *Machimosaurus* is known from the Guimarota site, Leiria, Portugal (Alcobaça Beds Formation) [6,7] (figures 4–10). The Guimarota site was deposited in either a lagoonal environment with some freshwater influx or a wooded swamp similar to extant mangrove forests. This locality has yielded several species of crocodylomorphs such as *Lusitanisuchus mitracostatus*, *Goniopholis baryglyphaeus* and *Theriosuchus guimarotae* [23,24].

**Figure 11.**
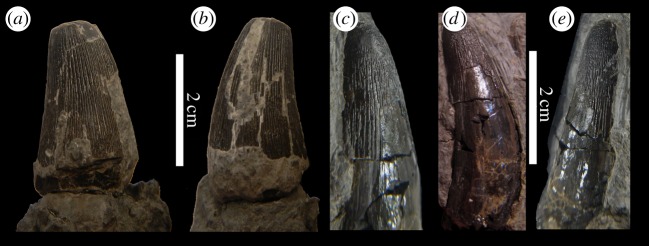
*Machimosaurus hugii*, MUJA-1298 and MUJA-1008, referred specimens. Isolated tooth crowns, (*a*,*b*) MUJA-1298 and (*c*–*e*) MUJA-1008.

From Spain:

— Isolated cf. *Machimosaurus* teeth have been reported from the Tereñes Formation of the Asturias coast, Northern Spain [25]. This formation is considered to represent a shallow tide-less sea [26]. Teeth are also known from the Lastres Formation in Asturias [25], a fluvial-dominated deltaic system in origin [26] (figure 11).
— The Kimmeridgian ichnogenus *Hatcherichnus* is known from coastal and deltaic units of Asturias, Spain. It has been suggested that these track ways were made by either *Machimosaurus* or a large goniopholidid [27].

**Figure 12.**
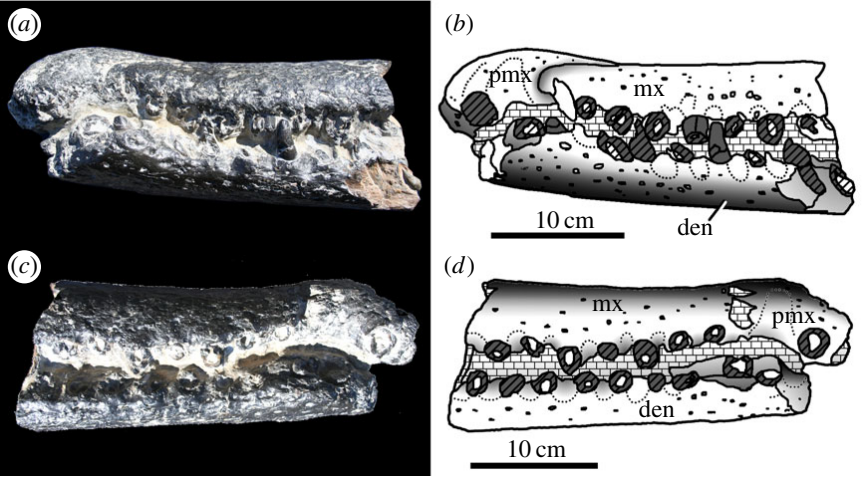
*Machimosaurus buffetauti* sp. nov., MPV V1600.Bo, referred specimen. Anterior region of the snout and lower jaw, (*a*) photograph in left lateral view, (*b*) line drawing in left lateral view, (*c*) photograph in right lateral view and (*d*) line drawing in right lateral view. den, dentary; mx, maxilla; pmx, premaxilla.

**Figure 13.**
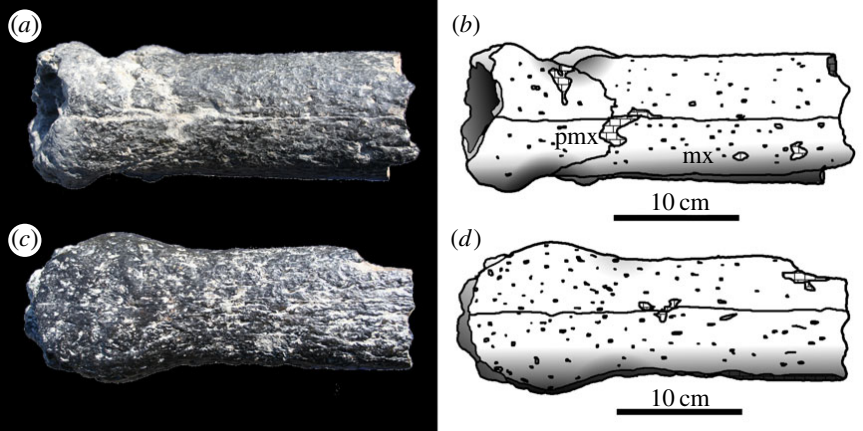
*Machimosaurus buffetauti* sp. nov., MPV V1600.Bo, referred specimen. Anterior region of the snout and lower jaw, (*a*) photograph in dorsal view, (*b*) line drawing in dorsal view, (*c*) photograph in ventral view and (*d*) line drawing in ventral view. mx, maxilla; pmx, premaxilla.

**Figure 14.**
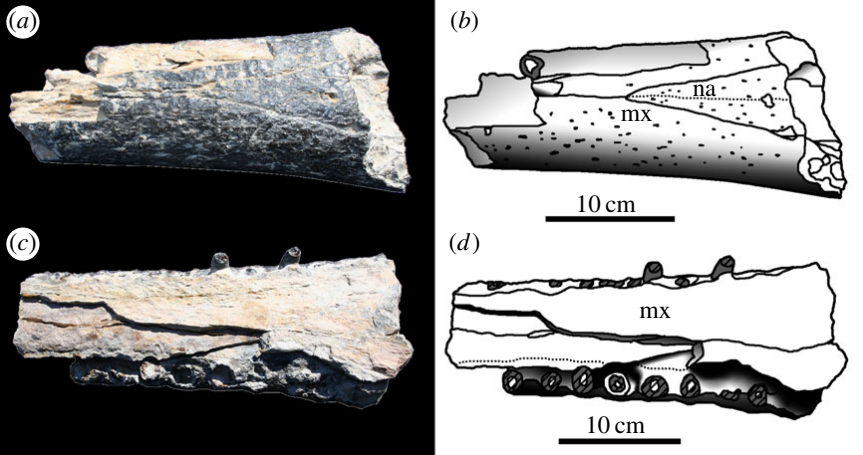
*Machimosaurus buffetauti* sp. nov., MPV V1601.Bo, referred specimen. Middle region of the snout, (*a*) photograph in dorsal view, (*b*) line drawing in dorsal view, (*c*) photograph in ventral view and (*d*) line drawing in ventral view. mx, maxilla; na, nasals.

**Figure 15.**
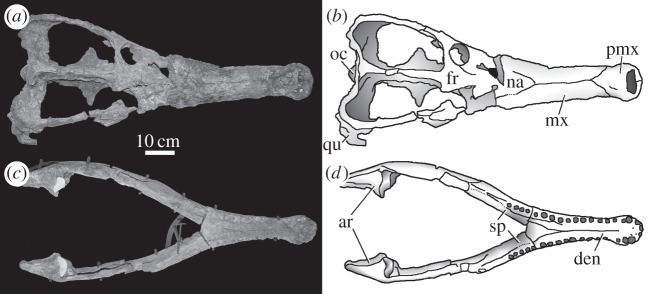
*Machimosaurus mosae*, neotype. Skull (*a*) photograph in dorsal view and (*b*) line drawing in dorsal view; mandible (*c*) photograph in dorsal view and (*d*) line drawing in dorsal view. ar, articular; den, dentary; fr, frontal; mx, maxilla; na, nasals; oc, occipital condyle; pmx, premaxilla; qu, quadrate; sp, splenial.

**Figure 16.**
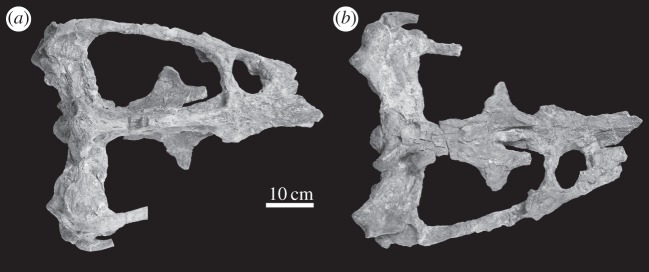
*Machimosaurus mosae*, neotype. Skull (orbital and temporal region) in (*a*) dorsal view and (*b*) ventral view.

**Figure 17.**
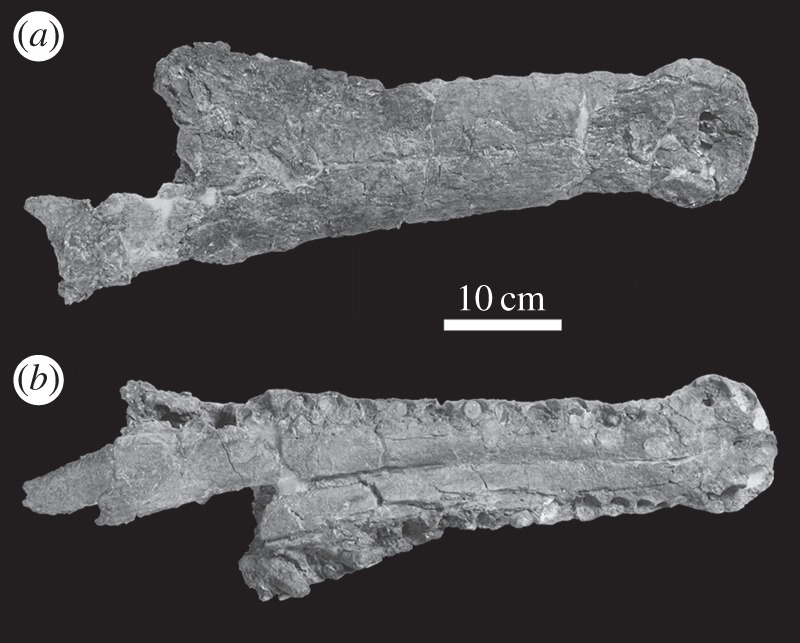
*Machimosaurus mosae*, neotype. Skull (rostrum) in (*a*) dorsal view and (*b*) ventral view.

**Figure 18.**
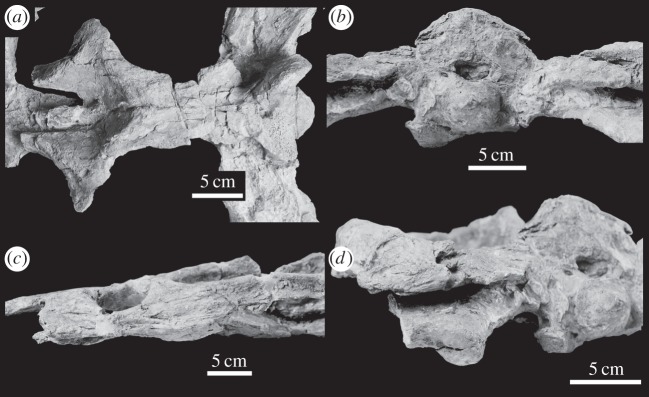
*Machimosaurus mosae*, neotype. Skull close-ups, (*a*) pterygoid, internal choana, basisphenoid and basioccipital in palatal view, (*b*) occipit in occipital/posterior view, (*c*) orbital region in left lateral view and (*d*) left quadrate, squamosal and paroccipital process region in lateral view.

**Figure 19.**
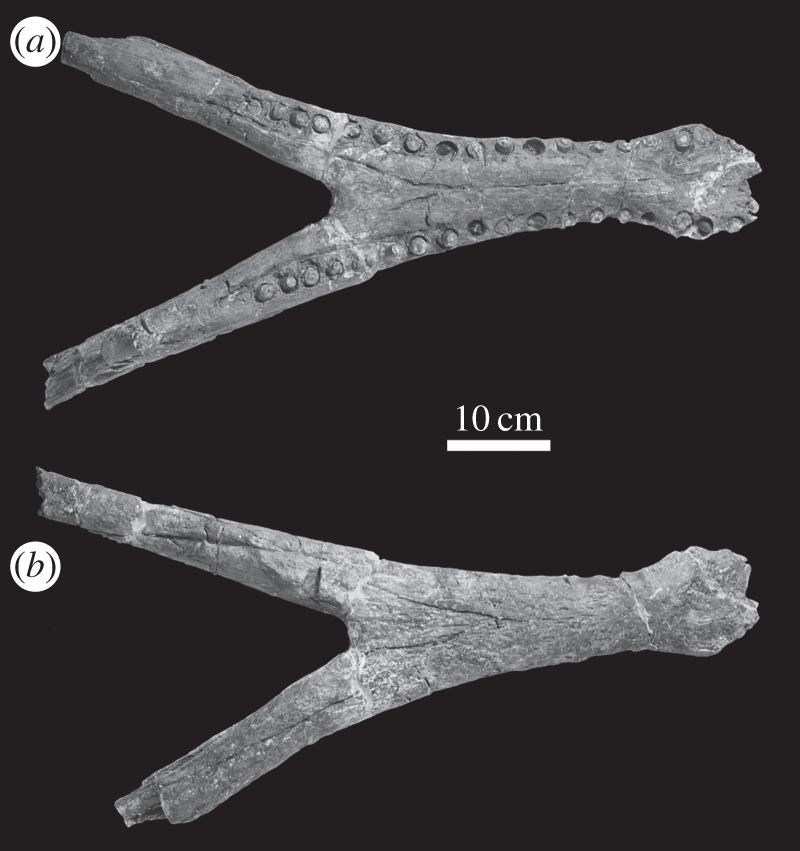
*Machimosaurus mosae*, neotype. Lower jaw (lacking the posterior ends of the rami) in (*a*) dorsal view and (*b*) ventral view.

**Figure 20.**
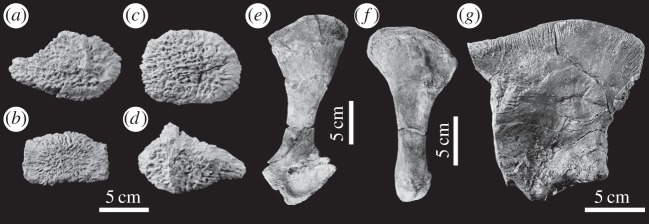
*Machimosaurus mosae*, neotype. Postcrania, (*a*) keeled ventral osteoderm, (*b*) ventral osteoderm, (*c*) dorsal osteoderm, (*d*) dorsal osteoderm, (*e*) right coracoid in medial view, (*f*) left pubis in medial view and (*g*) left ischium in lateral view.

From France:

— The anterior half of a rostrum and mandible in occlusion (premaxilla, maxilla and dentary) that has been attributed to *M. hugii* is known from the Calcaires Coquilliers Formation (*Pictonia baylei* Sub-Boreal ammonite zone, lowermost Kimmeridgian) of Cricqueboeuf, Normandy, Northern France [2,17] (figures 12–14). During the Early Kimmeridgian, the Calcaires Coquilliers Formation was deposited in a homoclinal mid-ramp with significant storm-wave reworking [28].
— The most complete skull of *M. hugii* was discovered from Ain, France (Lower Kimmeridgian) [3]. During the Kimmeridgian–Tithonian this region was a lagoonal environment [29].
— An almost complete skeleton of *M. mosae* was discovered near Ambleteuse, Boulonnais, France (Argiles de Châtillon Formation, either the *Aulacostephanus* *autissiodorensis* Sub-Boreal ammonite zone, uppermost Kimmeridgian or the *Gravesia gigas*/*Pectinatites elegans* Sub-Boreal ammonite zone, lowermost Tithonian) [4,21] (figures 15–20). The Argiles de Châtillon Formation was deposited in a nearshore or shallow-shelf marine environment off the west coast of the London–Brabant Massive [30].

**Figure 21.**
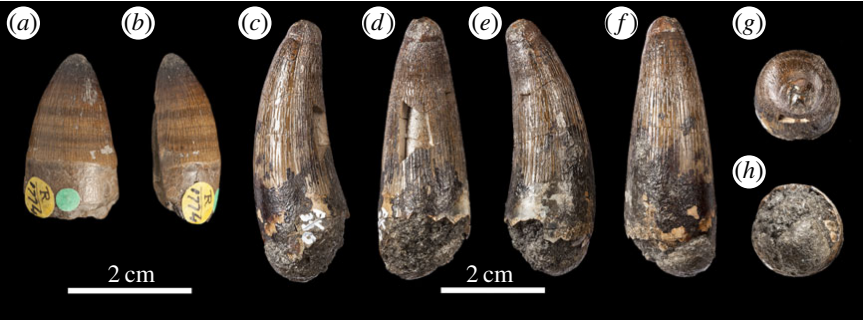
Isolated tooth crowns either referable, or likely to be referable to *M. buffetauti* sp. nov. NHMUK PV R1774 in (*a*) lingual view and (*b*) left lateral view; DFMMh FV 330 in (*c*) right lateral view, (*d*) lingual view, (*e*) left lateral view, (*f*) labial view, (*g*) apical view and (*h*) basal view.

**Figure 22.**
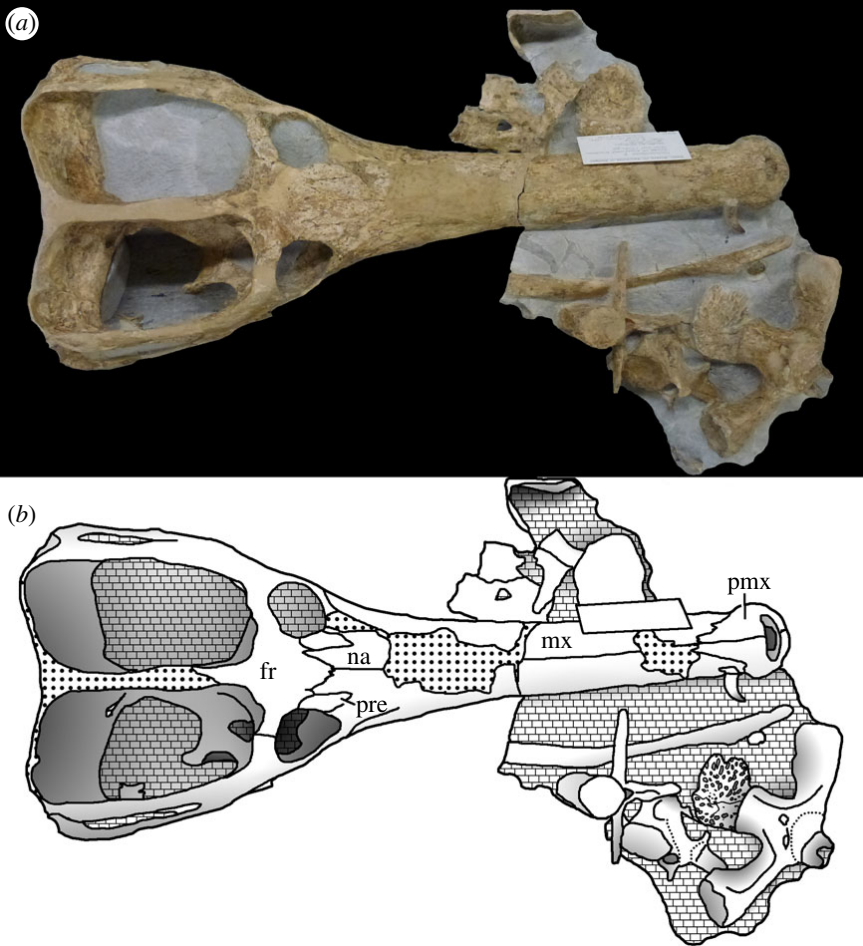
*Machimosaurus buffetauti* sp. nov., SMNS 91415, holotype. Skull (with associated postcrania) in dorsal view, (*a*) photograph and (*b*) line drawing. fr, frontal; mx, maxilla; na, nasals; pre, prefrontal; pmx, premaxilla.

**Figure 23.**
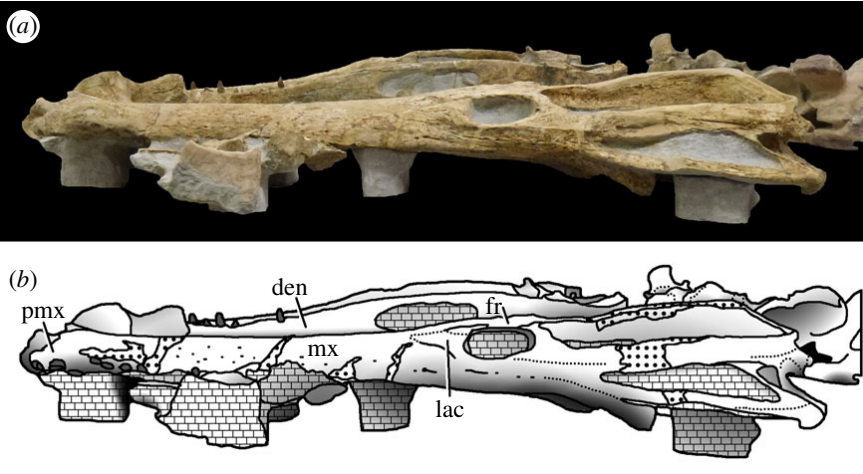
*Machimosaurus buffetauti* sp. nov., SMNS 91415, holotype. Skull (with lower jaw and associated postcrania) in right lateral view, (*a*) photograph and (*b*) line drawing. den, dentary; fr, frontal; lac, lacrimal; mx, maxilla; pmx, premaxilla.

**Figure 24.**
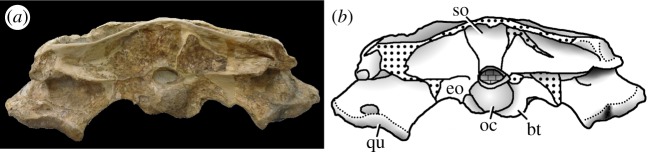
*Machimosaurus buffetauti* sp. nov., SMNS 91415, holotype. Skull in occipital view, (*a*) photograph and (*b*) line drawing. bo, basioccipital; bt, basioccipital tuberosities; eo, exoccipital; oc, occipital condyle; qu, quadrate; so, supraoccipital.

**Figure 25.**
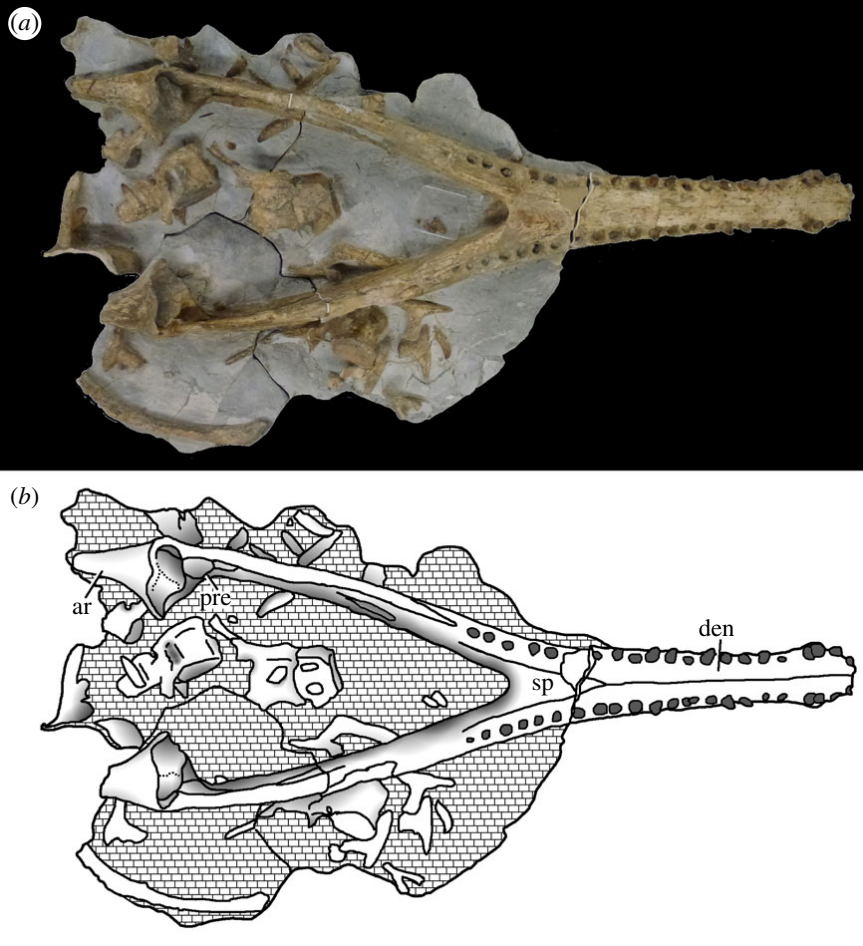
*Machimosaurus buffetauti* sp. nov., SMNS 91415, holotype. Lower jaw (with associated postcrania) in dorsal view, (*a*) photograph and (*b*) line drawing. ar, articular; den, dentary; pra, prearticular; sp, splenial.

**Figure 26.**
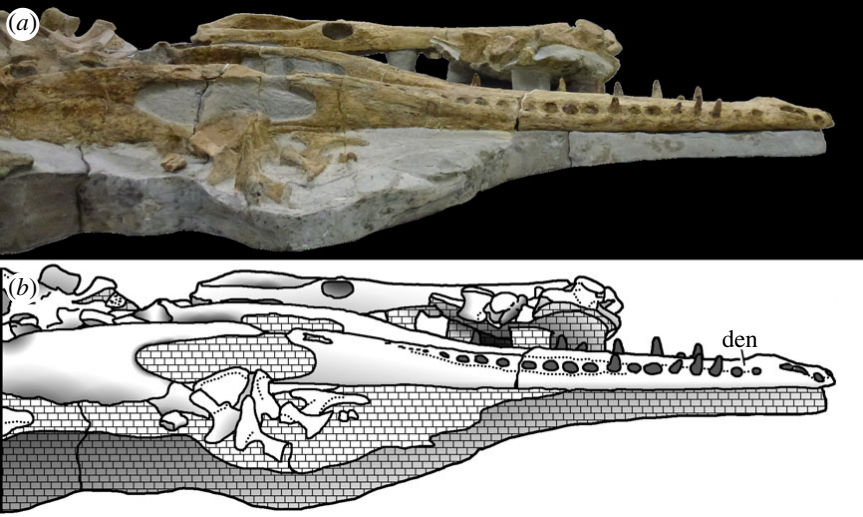
*Machimosaurus buffetauti* sp. nov., SMNS 91415, holotype. Lower jaw (with skull and associated postcrania) in right lateral view, (*a*) photograph and (*b*) line drawing. den, dentary.

**Figure 27.**
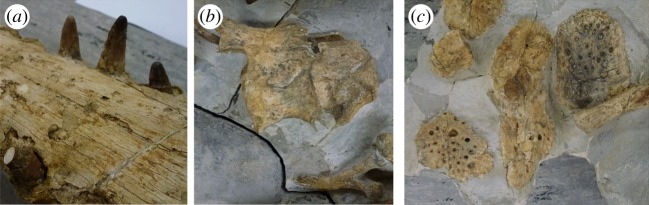
*Machimosaurus buffetauti* sp. nov., SMNS 91415, holotype. (*a*) Close-up on the dentary dentition, (*b*) the atlas–axis in lateral view and (*c*) the dorsal osteoderms in dorsal view.

From Germany:

— von Meyer [31] referred a tooth from Kahlenberg, near Hannover, in Lower Saxony to *Machimosaurus*. This locality is now within the urban area of Hannover [32].
— Numerous isolated teeth have been discovered at the Oker quarry, Langenberg area (Langenberg Formation) of Lower Saxony [8,32] (figure 21*c*–*h*). Sediments from this area were deposited in a shallow-water basin, either a bay or a lagoon, and, along with *Machimosaurus*, numerous other crocodylomorphs are known from this region: *Goniopholis simus*, *Theriosuchus pusillus*, *Steneosaurus* sp. and two gen. et sp. nov. [32].
— A skull, lower jaw and partial postcranial skeleton of *M. hugii* was discovered in a quarry at Neuffen, Baden-Württemberg (*Ataxioceras hypselocyclum* Sub-Mediterranean ammonite zone, Weißer Jura gamma 2, Lower Kimmeridgian) [10] (figures 22–27).
— *Machimosaurus* has also been reported from Fritzow, Mecklenburg-Vorpommern, Germany [33].

**Figure 28.**
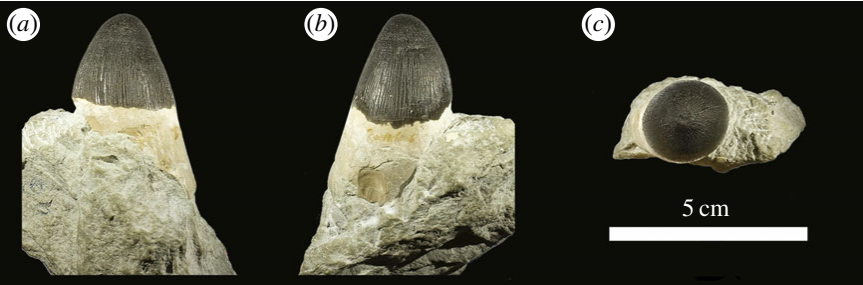
*Machimosaurus hugii*, NMS 8342, lectotype. Isolated tooth crown in (*a*) labial view, (*b*) lingual view and (*c*) apical view.

**Figure 29.**
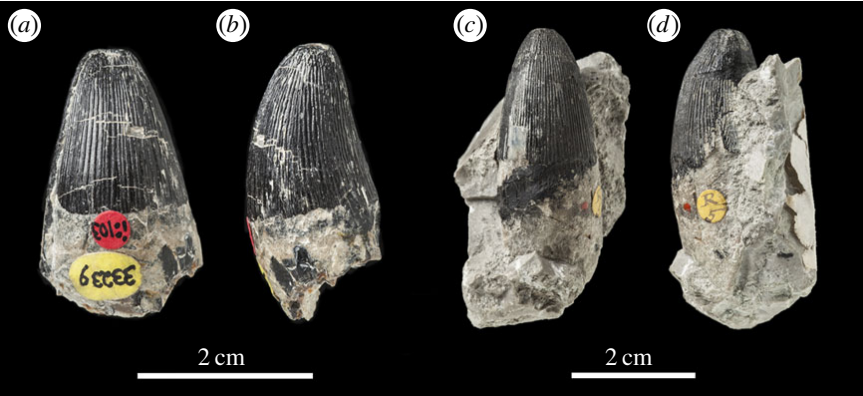
*Machimosaurus hugii* referred specimens. NHMUK PV OR33239 in (*a*) labial view and (*b*) right lateral view; NHMUK PV R5 in (*c*) labial view and (*d*) right lateral view.

**Figure 30.**
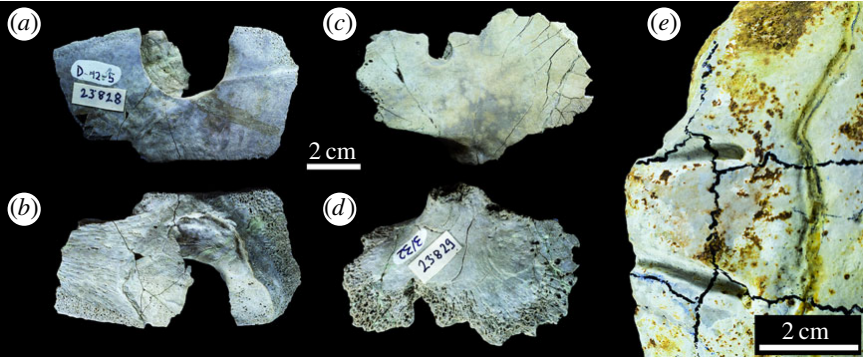
Marine turtle specimens from the Solothurn Turtle Limestone of Switzerland with *Machimosaurus* bite marks. All are from Steinbruch, Solothurn. Plastron fragments of Testudinata indet. NMS 23828 in (*a*) dorsal view and (*b*) ventral view. Plastron fragments of NMS 23829 in (*c*) dorsal view and (*d*) ventral view. (*e*) Carapace of *Plesiochelys* sp. NMS 21499.

From Switzerland, *Machimosaurus* material is known from:

— A dorsal vertebra attributed to *M*. *hugii* from Moutier, Canton Bern (possibly Early Kimmeridgian) [34]. In addition, a broken sauropod dinosaur (*Cetiosauriscus greppini*) femur has bite marks matching *Machimosaurus* teeth [34]. Interestingly, it has been suggested that these specimens were buried in freshwater sediments due to the greenish marl [34].
— Isolated teeth are known from the ‘Solothurn Turtle Limestone’ (uppermost part of the Reuchenette Formation, Late Kimmeridgian, *Hybonoticeras* *beckeri* Sub-Mediterranean ammonite zone [35]) of Solothurn, Canton Solothurn (figures 28 and 29). Marine turtle shells (Plesiochelyidae) discovered from this limestone are known to have bite marks matching *Machimosaurus* teeth, and in some instances still have *Machimosaurus* teeth imbedded within them [36] (figure 30). The ‘Solothurn Turtle Limestone’ is interpreted as being a shallow protected lagoon [37]. The type series (isolated tooth crowns) of *M. hugii* were found in this limestone [6,7,31].
— A second sea turtle assemblage found in the Virgula Marls near Porrentruy, Canton Jura (Late Kimmeridgian, *A*. *eudoxus* Sub-Mediterranean ammonite zone [35]) also has yielded isolated *Machimosaurus* teeth [38].

**Figure 31.**
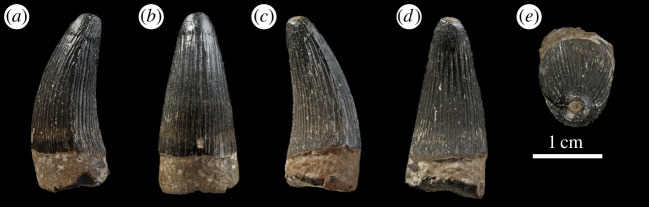
*Machimosaurus* cf.*buffetauti*, GPIT/RE/9280. Isolated tooth crown in (*a*) right lateral view, (*b*) labial view, (*c*) left lateral view, (*d*) lingual view and (*e*) apical view.

**Figure 32.**
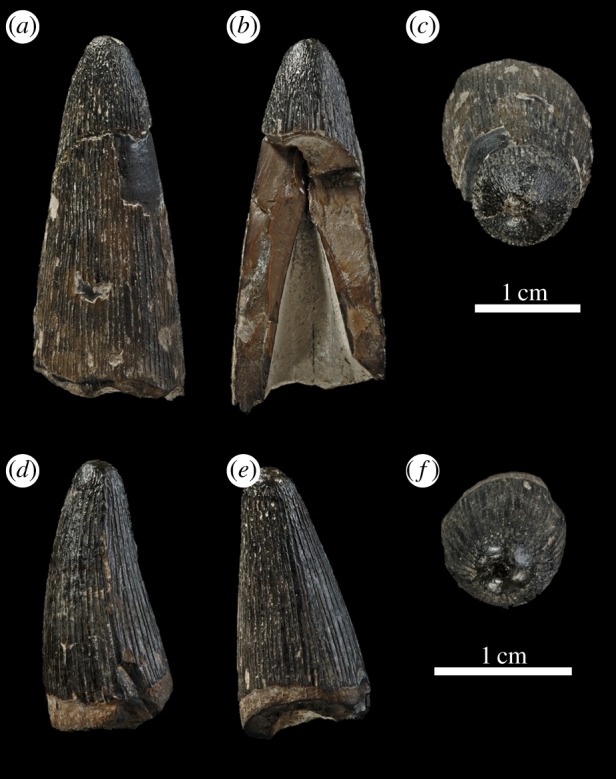
*Machimosaurus* cf. *buffetauti*. Isolated tooth crown GPIT/RE/328 in (*a*) lingual view, (*b*) labial view and (*c*) apical view. Isolated tooth crown GPIT/RE/9281 in (*d*) right lateral view, (*e*) labial view and (*f*) apical view.

From Poland, isolated *Machimosaurus* teeth are known from Lower Kimmeridgian deposits of the Czarnogłowy quarry, West Pomerania (note that prior to 1945 Czarnogłowy was in Germany and was called Zarnglaff, and that this name is used in pre-1945 literature) [6,39,40] (figures 31 and 32). Dzik [40, fig. 9.20C] figured a mandibular fragment (symphyseal region lacking the anterior-most half/third) from Czarnogłowy as *Machimosaurus*. However, based on comparisons between *Steneosaurus* and *Machimosaurus* specimens, this partial mandible is in fact *Steneosaurus*, as: (i) it has a proportionally narrow mandible with a high tooth count; (ii) the anterior-most preserved alveoli have inter-alveolar spaces which are too long, being greater than the length of the adjacent alveoli; (iii) the splenial is very elongated and has at least 16 pairs of symphyseal alveoli adjacent; and (iv) the Meckelian groove is very deeply excavated, especially at the mandibular midline [1,4,10,14].

From England,*Machimosaurus* is solely known from a single incomplete isolated tooth crown discovered at Smallmouth Sands, Dorset (Lower Kimmeridge Clay Formation) [5, fig. 215A,B]. Until recently, *M. mosae* was considered to be present in the Upper Kimmeridge Clay Formation of England (Early Tithonian). This was based on a very large skull and mandible from Kimmeridge in Dorset, which was recently shown to pertain to the metriorhynchid crocodylomorph *Plesiosuchus manselii* [41]. This means that the single tooth crown from Smallmouth Sands is the only *Machimosaurus* specimen known from England. Although the Dorset succession of the Kimmeridge Clay Formation is considered to have formed at an outer-shelf water depth of between 150 and 200 m [42], during the Early Kimmeridgian the water depth was very shallow in the Dorset succession, between 10 and 30 m [43].

### 3.6 The Kimmeridgian–Tithonian boundary

**Figure 33.**
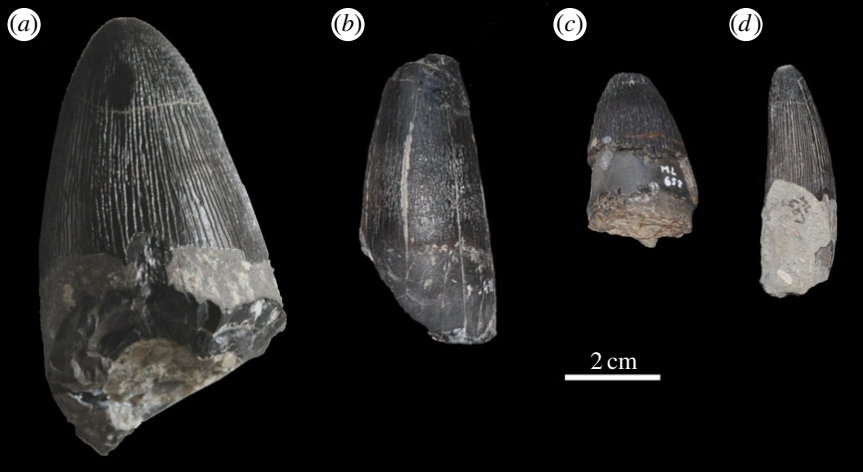
*Machimosaurus hugii*, referred specimens. Isolated tooth crown (*a*) ML 491 in labial view, (*b*) ML 657 in labial view, (*c*) ML 658 in labial view and (*d*) ML 657 in lingual/lateral view.

**Figure 34.**
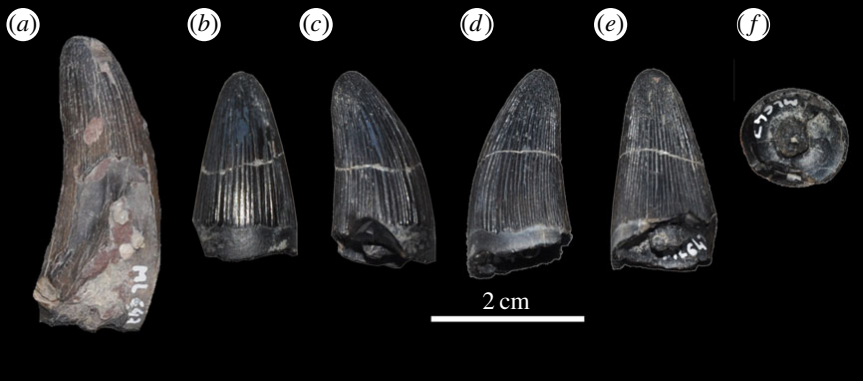
*Machimosaurus hugii*, ML 647, referred specimens. First isolated tooth crown in (*a*) left lateral view. The second tooth crown in (*b*) labial view, (*c*) left lateral view, (*d*) right lateral view, (*e*) lingual view and (*f*) basal view.

**Figure 35.**
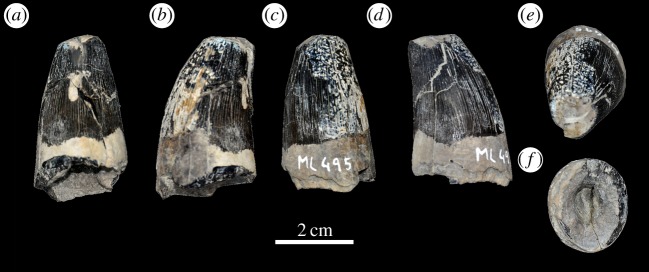
*Machimosaurus hugii*, ML 495, referred specimen. Isolated tooth crown in (*a*) labial view, (*b*) right lateral view, (*c*) lingual view, (*d*) left lateral view, (*e*) apical view and (*f*) basal view.

All *Machimosaurus* specimens from the Lourinhã Formation in Portugal are from the Praia Azul Member, which was a brackish to coastal platform that comprised the Kimmeridgian–Tithonian boundary at 152.1 Ma [44,45]. However this can be better dated as *ca* the Upper Kimmeridgian–Lower Tithonian transition. The rest of the Lourinhã Formation extends to the Jurassic–Cretaceous boundary, but the sediments are strictly continental (flood-plain mudstones and fluvial sandstone bodies [46]). Thus, the absence of *Machimosaurus* in the Tithonian of Portugal is solely due to a shift in palaeoenvironment, rather than a true disappearance of the genus in the Tithonian. Sauvage [18] reported an isolated tooth from the Upper Kimmeridgian–Lower Tithonian of Santa-Cruz (Praia Azul Member, Lourinhã Formation [44]). New discoveries in the Lourinhã area include: several isolated teeth, all from the Praia Azul Member (*sensu* [44]), from the following localities: Porto das Barcas (ML491, ML495, ML959 and ML1955), Peralta (ML647), Zimbral (ML657 and ML658) and around coastline (ML647, ML733 and ML902) (figures 33 and 34). Some of these tooth crowns are very large in size: the tooth ML495 from Porto das Barcas (N39°13.943′; 9°20.349′) has an apicobasal length of 41.4 mm (crown alone is 27.12 mm) and maximal diameter of 24.2 mm (figure 35).

A large vertebra previously referred to cf. *Machimosaurus* [47] is here no longer regarded as a crocodylomorph. The Lourinhã Formation has a rich and diverse dinosaur fauna, including eggs and tracks, but crocodylomorph remains are also common, mostly *Goniopholis* and *Theriosuchus*.

### 3.7 Tithonian

An isolated *Machimosaurus* tooth is known from marine deposits of the Higueruelas Formation at Buñol, Valencia Province, Eastern Spain [48].

Sauvage [11, planche 3] stated that the holotype (‘type de l'espèce’) of *M. interruptus* is from the ‘Portlandien à *Ammonites portlandicus* de Mont-Lambert (near Boulogne-sur-Mer, France)’. As *Ammonities portlandicus* is a synonym of *G. gigas* [11], it places this tooth in the Early Tithonian *G. gigas*/*P. elegans* Sub-Boreal ammonite zone.

### 3.8 Berriasian

Isolated teeth from Spain, with a possible Berriasian age, have previously been attributed to *Machimosaurus*. In 1916–1918, JM Catalá discovered a series of fossil vertebrates from Benagéber, Valencia province, Spain. The description of Beltrán [49] stated that the fossils were from the ‘Wealdense’ (i.e. Wealden facies) and that *Gonophilus* (sic) teeth were among the collection. Royo y Gómez [50] reviewed the Catalá collection when it was temporarily loaned to the Museo Nacional de Ciencias Naturales in Madrid. Two species of crocodylomorphs were reported as being present: *Goniopholis* and *Machimosaurus*. One year later, Royo y Gómez assigned the Benagéber crocodiles to*Steneosaurus* cf. *obtusidens* and *Machimosaurus* sp. nov. [51], specifying its age as ‘Purbequiense’ (i.e. Purbeck facies) [52]. Therefore, it is possible that the teeth were Tithonian or Berriasian in age.

Unfortunately, these teeth were never described or figured and are currently missing. It is possible that they were destroyed in the fire that ruined the Museo de Historia Natural de la Universidad de Valencia in 1932, were Beltrán was a professor [53]. As such, the presence of *Machimosaurus* in the earliest Cretaceous of Spain cannot currently be confirmed.

### 3.9 Valanginian

A partial dentary with *in situ* tooth crowns from the Valanginian of southern France (Département des Bouches-du-Rhône) was described as *Steneosaurus* sp. due to a superficial similarity in dental morphology between it and ‘*S*.’ *obtusidens* [54]. However, recent re-examination of the Valanginian specimen demonstrated it to be a metriorhynchid closely related to *P. manselii* [55].

### 3.10 Upper Hauterivian–Lower Barremian

Sanz *et al.* [56] referred isolated tooth crowns from the Lower Cretaceous of Galve (Teruel province, Spain) to cf. *Machimosaurus* sp. These teeth come from the sediments on the top of El Castellar Formation (uppermost Hauterivian–lowermost Barremian), which is lacustrine in origin [57]. Intriguingly, *Machimosaurus* was not cited in the subsequent papers on the Galve crocodylomorphs made by the same authors (e.g. [58,59]), indicating that they may have been hesitant in this taxonomic assignment.

The teeth were described as being: ‘distinguished by blunt crowns with a very characteristic enamel ornamentation of anastomosed and braided ridges’ [52, p. 207]. One of the teeth was figured [56, p. 208, fig. 3*a*,*b*], and based on that line drawing we do not consider it referable to *Machimosaurus*. This is due to the tooth's strong lingual curvature, concave lingual surface and strongly pronounced mesial and distal carinae, all characteristics not seen among *Machimosaurus* teeth.

Gasca *et al.* [60] mentioned aff. *Machimosaurus* teeth in the Lower Cretaceous of Allepuz (Teruel province, Spain). These teeth came from a microvertebrate site in the Camarillas Formation (Lower Barremian) that originated in an avulsion deposit, namely an ephemeral fluviatile pond, and presents a mixture of terrestrial (theropod dinosaurs and crocodylomorphs: Bernissartiidae, Atoposauridae, aff. *Machimosaurus*) and freshwater vertebrates (hybodontid sharks, bony fishes and amphibians). Unfortunately, these teeth have never been described or figured, thus we cannot determine the reliability of this taxonomic assessment. Therefore, there is no evidence that *Machimosaurus*, or any other teleosaurid, survived into the Cretaceous.

## 4. Recent taxonomic changes to *Machimosaurus hugii*

### 4.1 The diverse and long-lived *Machimosaurus hugii*

Recently, Pierce *et al*. [9] proposed that *M. hugii* was the senior subjective synonym of various Callovian teleosaurids: ‘*S*.’ *obtusidens*, *Steneosaurus durobrivensis* and *Steneosaurus hulkei*. No evidence for this taxonomic revision was given. However, they considered *M. mosae* to be distinct from this long-lived (more than 10 Ma) *M. hugii* species. Martin & Vincent [10, p. 194] criticized the content of their species diagnoses, as: ‘most of the content of these diagnoses reveal to be either diagnostic at the genus level or to characterize all Teleosauridae’. Martin & Vincent [10, pp. 194–195] went on to show that the very high variation in maxillary and dentary tooth counts among the various Callovian teleosaurids is: ‘sufficient difference to discard such an interpretation (the synonymy)’. We concur with this assessment. Below, we propose a revised diagnosis for *Machimosaurus*, which has numerous autapomorphies absent in these Callovian species. Moreover, multiple phylogenetic analyses falsify the synonymy of *M. hugii* with *S. durobrivensis* and/or ‘*S*.’ *obtusidens* [10,41,61].

### 4.2 The diverse Kimmeridgian *Machimosaurus hugii*

Recently, Martin & Vincent [10] described an incomplete skeleton of *Machimosaurus* from the Lower Kimmeridgian of Germany. They referred this specimen to *M. hugii*, along with all other Kimmeridgian *Machimosaurus* specimens from Europe. This included synonymizing *M. mosae* with *M. hugii*. However, they did not discount that a second taxon could be determined based upon further investigation of relevant specimens [10, p. 193].

This paper reopened an old debate about whether *Machimosaurus* is a monotypic genus, and whether the differences between *M. hugii* and *M. mosae* are due to ontogeny. This issue has been examined in detail by Hua [4] and Vignaud (1995, unpublished PhD thesis), who studied teleosaurid ontogeny using the numerous European skulls available. Both of these authors considered *M. mosae* to be taxonomically distinct from *M. hugii*. We reject the hypothesis that the *M. mosae* neotype is a juvenile of *M. hugii* below, based on four fundamental flaws in this ‘juvenile hypothesis’: (i) the *M. mosae* neotype is comparable in size to the French [3] and German [10] skulls referred to *M. hugii*, all three of which differ in length by only 7 cm (93–100 cm; table 1); (ii) the lack of juvenile characteristics in any of the French [3,4] and German [10] skulls [58]; (iii) the *M. mosae* neotype has exostoses (formation of new bone on the surface of bones, usually seen in mature individuals) in the femur, right pubis and on the transverse processes of some caudal vertebrae [4]; and (iv) the *M. mosae* neotype is from the uppermost Kimmeridgian, whereas the two skulls referred to *M. hugii* are from the Lower Kimmeridgian, a temporal gap of some 3–5 million years.

**Table 1.** Comparison of biometric variation among well-preserved *Machimosaurus* specimens.

species	*M. buffetauti*		*M. mosae*		*M. hugii*
reference	[10]	[3]	[62]	[4]	[6]
basicranial length (cm)	93.5	100	approx. 130	96.5	approx. 149
rostrum length (cm)	54.7	58	72^*b*^	56.2	?
ratio of rostrum length to basicranial length (%)	58.5	58	55^*c*^	58.2	?
maximum width of the skull (cm)	39.7	33^*a*^	58	43	?
ratio of maximum skull width to basicranial length (%)	42.5	33^*a*^	44.6	44.6	?
maximum supratemporal fenestra length (cm)	26	27.5	∼40	32.2	?
ratio of maximum supratemporal fossa length to basicranial length (%)	27.8	27.5	30.8	33.4	?
length of mandible (cm)	95.4	?	132.5^*b*^	112	?
length of mandibular symphysis (cm)	48.6	?	62^*b*^	47.5	?
ratio of symphysis length to mandible length (%)	50.9	?	46.8^*b*^	42.4	?

^*a*^Much of the skull has been reconstructed with plaster, making it difficult to discern what is bone and what is plaster ([3] and E. Buffetaut 2014, personal communication).

^*b*^Estimate [62].

^*c*^The basicranial and rostrum length estimates of Sauvage & Liénard [62] are most likely slight underestimates. From the skull line drawing in plate 1 fig. 1, it looks like the rostrum is too close to the orbital region of the skull. As such, the skull was probably mesorostrine (ratio of rostrum length to basicranial length would have been slightly more than 55%).

Moreover, there is another character that shows that *M. mosae* is a distinct taxon from *M*. *hugii*, the presence of the prearticular (figure 25). Martin & Vincent [10] described the first prearticular ever mentioned for a teleosaurid (although they did not highlight its significance). Prearticular bones were previously only known in Metriorhynchidae among thalattosuchians [1]. Interestingly, and most importantly for *Machimosaurus* systematics, the prearticulars are not found in the mandible of *M. mosae* [4]. Therefore, the loss of the prearticulars is a specific character for *M. mosae* (as the prearticulars are also present in *S. larteti*; figure 1*a*,*b*). (Owing to the status of the *M. mosae* neotype, we cannot currently test the hypothesis whether the absence of the prearticulars is a preservational artefact.)

**Figure 36.**
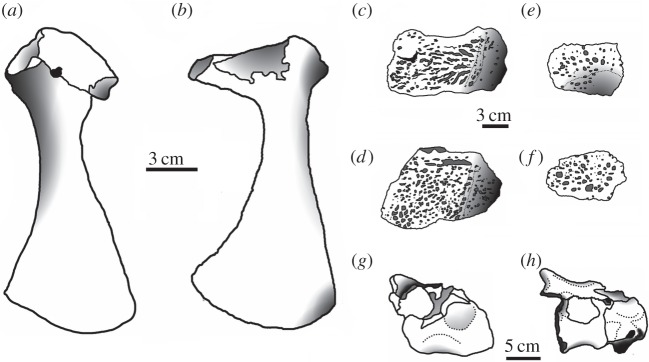
Postcranial element comparison between the holotype of *M. buffetauti* and the neotype of *M. mosae* (based on the figures in [4,10]). Coracoids of (*a*) *M. mosae* and (*b*) *M*. *buffetauti*; the dorsal osteoderms of (*c*,*e*) *M*. *mosae* and (*d*,*f*) *M*. *buffetauti*; the atlas–axis of (*g*) *M. mosae* and (*h*) *M. buffetauti*.

Postcranial characteristics supporting the distinction of *M*. *hugii* and *M*. *mosae*—not examined by Martin & Vincent [10], who unfortunately mainly focused on the skull—include:

— Coracoids are highly variable among teleosaurid species [1]. The same is true between the new specimen of *M*. *hugii* and *M. mosae*, especially in the shape and size of the postglenoid and glenoid processes (figure 36*a*,*b*). In the German *M*. *hugii* skull [10], the coracoid glenoid process (process near the glenoid fossa that projects posterodorsally) is elongate, extending considerably from the head of the coracoid, and is a sub-isosceles triangle in shape when seen in lateral view; the coracoid postglenoid process anterior margin is very slightly concave and terminates approximately in the samef frontal plane as the glenoid; and the postglenoid process posterior margin is strongly concave and terminates approximately in the same frontal plane as the posterior-end of the glenoid process. However in the *M. mosae* neotype [4], the coracoid glenoid process is very short, not extending far from the head of the coracoid, and is a right-angled triangle in shape when seen in lateral view; the coracoid postglenoid process anterior margin is strongly concave and terminates in a frontal plane anterior to the glenoid; and the postglenoid process posterior margin is strongly concave distally but shifts to being somewhat convex proximally and terminates in a frontal plane posterior to the posterior-end of the glenoid process.
— The difference in axis neural arch shape between their new specimen of *M*. *hugii* and *M. mosae* (figure 36*g*,*h*). In the German *M. hugii* specimen [10], the axis neural arch has a strongly concave dorsal margin and the postzygapophyses terminate notably posterior to the posterior surface of the centrum (figure 36*h*), whereas in the *M. mosae* neotype [4] the dorsal margin is only weakly concave and the postzygapophyses are not as long posteriorly (figure 36*g*). Compare Martin & Vincent [10, p. 191, fig. 9*a*–*c*] with Hua [4, plate 3 fig. 1–3].

Thanks to the new specimen described by Martin & Vincent [10] there are now numerous postcranial characteristics, along with the absence of the prearticular (assuming that it is not a preservational artefact), that allow easy identification of *M. mosae* and differentiate it from *M*. *hugii*. Furthermore, they described a depression on the dorsal surface of the quadrates near the hemicondyles on the German *M. hugii* skull. These depressions are not seen in *M. mosae* and comprise another feature differentiating these two species [4].

## 5. Description of *Machimosaurus hugii* by von Huene [63] and Krebs [6,7]

*Machimosaurus* teeth have long been known, particularly from the Kimmeridgian of Solothurn, Switzerland. One such tooth was figured by Cuvier in 1824 [64, plate 6 fig. 7]. In 1836, Römer [65] figured a *Machimosaurus* tooth from Kahlenberg, Germany, although he considered it to be *Ichthyosaurus* [65, p. 12, plate 12 fig. 19]. It was not until 1837 that the binomial *M. hugii* was specifically established for the Solothurn and Kahlenberg teeth [31]. Unfortunately, the name was misspelt as *Madrimosaurus hugii* in that publication [31], something von Meyer attributed to: ‘Die Undeutlichkeit meiner Handschrift’—‘the indistinctness of my handwriting’ [66, p. 415]. As such, von Meyer corrected the spelling in an 1838 publication [66].

**Figure 37.**
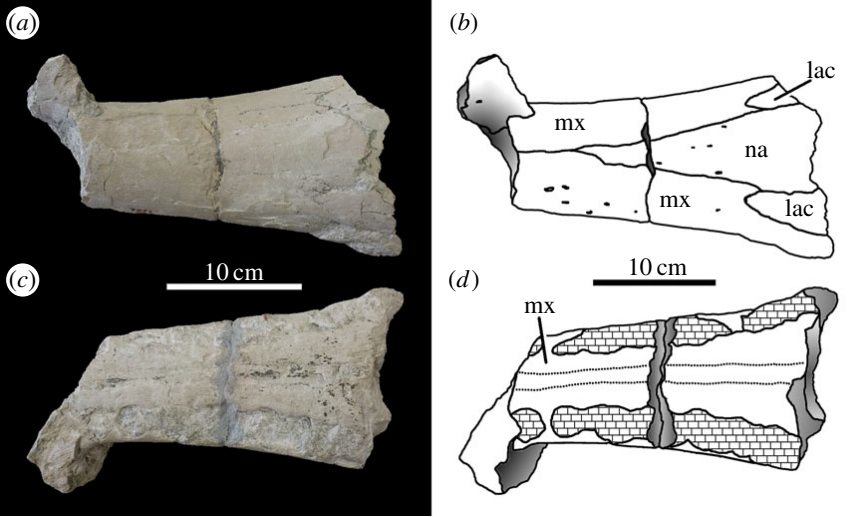
*Machimosaurus hugii*, NMS 7012, referred specimen. Incomplete snout (fragment consisting of the lacrimals, nasals and maxilla), (*a*) photograph in dorsal view, (*b*) line drawing in dorsal view, (*c*) photograph in ventral view and (*d*) line drawing in ventral view. lac, lacrimal; mx, maxilla; na, nasals.

**Figure 38.**
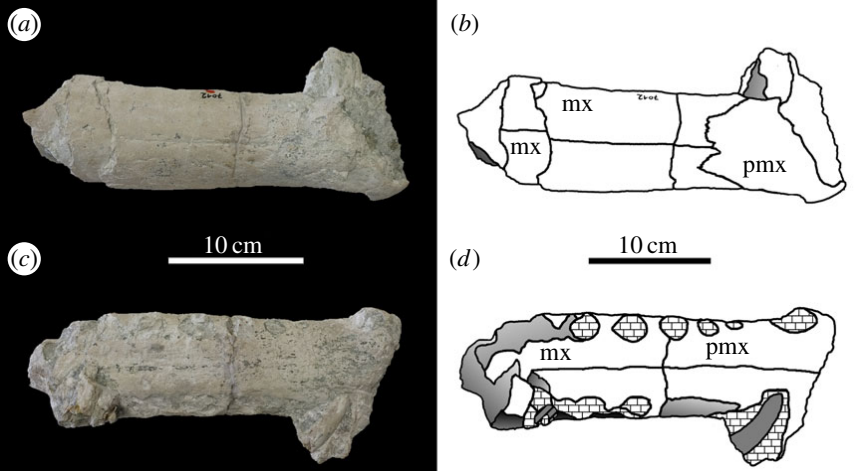
*Machimosaurus hugii*, NMS 7012, referred specimen. Incomplete snout (fragment consisting of the maxilla and premaxilla), (*a*) photograph in dorsal view, (*b*) line drawing in dorsal view, (*c*) photograph in ventral view and (*d*) line drawing in ventral view. mx, maxilla; pmx, premaxilla.

**Figure 39.**
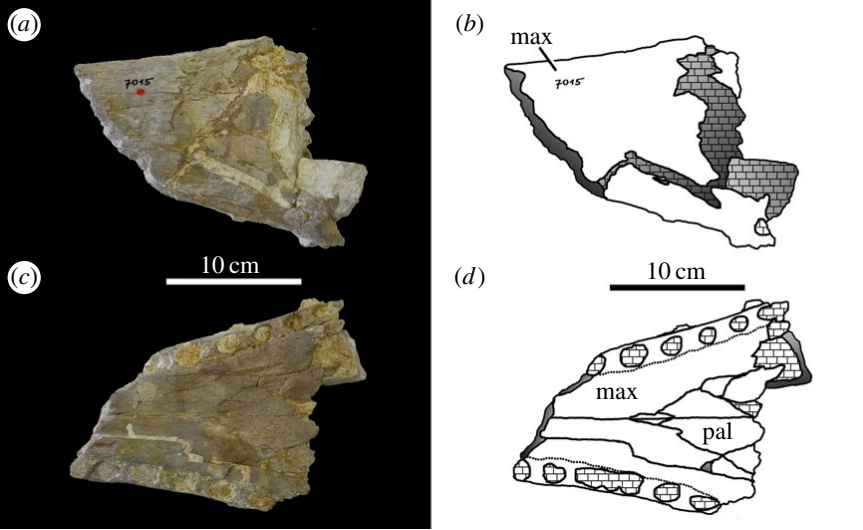
*Machimosaurus hugii*, NMS 7015, referred specimen. Incomplete snout (fragment consisting of the maxilla and palatines, damage makes determining other bones difficult), (*a*) photograph in dorsal view, (*b*) line drawing in dorsal view, (*c*) photograph in ventral view and (*d*) line drawing in ventral view. mx, maxilla; pal, palatines.

**Figure 40.**
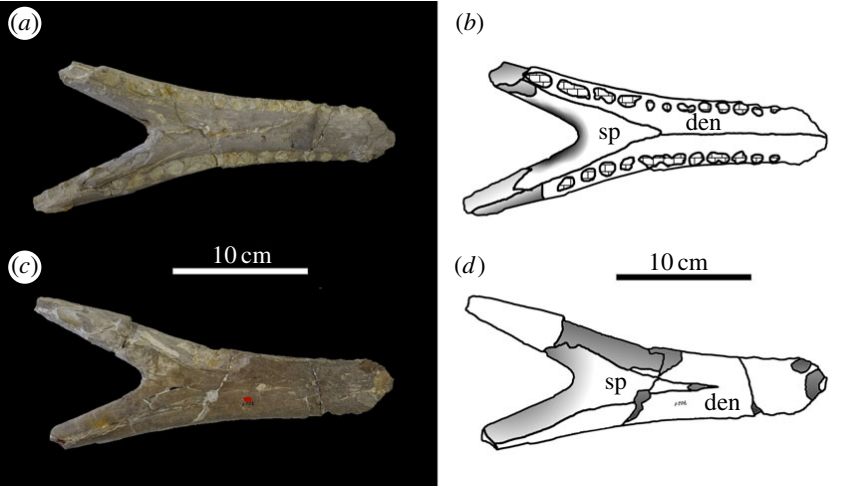
*Machimosaurus hugii*, NMS 7021, referred specimen. Incomplete lower jaw, (*a*) photograph in dorsal view, (*b*) line drawing in dorsal view, (*c*) photograph in ventral view and (*d*) line drawing in ventral view. den, dentary; sp, splenial.

Curiously, throughout all the various competing arguments over specimen synonymies regarding *M. hugii*, the description and figures of von Huene [63] and Krebs [6,7] (as well as the Solothurn teeth) depict a taxon which is distinct from the *M. mosae* and the ‘*M. hugii*’ specimens described during the 1980s—2010s from France and Germany. This taxon is known from Swiss and Portuguese material. The distinctiveness of this taxon relative to other *Machimosaurus* specimens has not been clearly recognized until now. As we show below, this taxon is *M*. *hugii*.

von Huene [63] described and figured a number of fragmentary skull and mandibular fragments from Switzerland that belong to this taxon (NMS 7012, NMS 7015 and NMS 7021; figures 37–40). His figures demonstrate five autapomorphies among *Machimosaurus* (and other teleosaurids): (i) the external surfaces of the snout bones are poorly ornamented with low relief ridges mostly orientated anteroposterly; (ii) sub-globidont dentition (blunt apices, low apicobasal height to basal width ratio, but the teeth lack the pronounced ‘globular’/bulbous morphology of true globidonty); (iii) apicobasally aligned enamel ridges immediately adjacent to the apical anastomosed region that are closely packed on both the labial and lingual tooth surfaces; (iv) uniform inter-alveolar spaces in the posterior–mid region of the maxillae, with the inter-alveolar spaces becoming slightly larger anteriorly but still being largely uniform in size; and (v) uniformly narrow inter-alveolar spaces in the dentaries. These characteristics are distinct from the morphologies seen in the *M. mosae* neotype [4,21] and the French and German ‘*M*. *hugii*’ specimens [2,3,10,17] (figures 12–14 and 21–27), in which: (i) the external surfaces of the snout bones are more strongly ornamented, with higher relief ridges and sub-circular/oval pits; (ii) no tooth crowns are sub-globidont; (iii) apicobasally aligned enamel ridges immediately adjacent to the apical anastomosed region are closely packed only on the lingual tooth surface, but on the labial surface these ridges are more widely spaced; (iv) the maxillary and (v) dentary inter-alveolar spaces are variable in size, some of which can be quite large proportionaly to the adjacent alveoli.

Krebs' [6,7] description and figures of the large, but fragmentary, Leiria skull from Portugal (MG-8730-1 and MG-8730-2; figures 4–9) reveal seven autapomorphies: (i) the external surfaces of the snout bones are poorly ornamented with low relief ridges mostly orientated anteroposterly; (ii) sub-globidont dentition; (iii) apicobasally aligned enamel ridges immediately adjacent to the apical anastomosed region that are closely packed on both the labial and lingual tooth surfaces; (iv) paroccipital processes that are greatly enlarged, both elongated mediolaterally and with lateral ends that are expanded; (v) basioccipital tuberosities (basal tubera) that are very large in size and are sub-rectangular in shape when seen in occipital view; (vi) the inter-basioccipital tubera notch is a large inverse ‘U’-shape when seen in occipital view; and (vii) uniformly spaced inter-alveolar spaces in the mid region of the maxillae. Moreover, the apicobasal ridge characteristic is also seen in the lectotype of *M. hugii* [6,7]. Once again, these characteristics are distinct from the morphologies seen in the *M. mosae* neotype [4,21] and the French and German ‘*M*. *hugii*’ specimens [2,3,10,17].

Even though these Swiss and Portuguese specimens are fragmentary, they share four autapomorphies that are not seen in any other teleosaurid: (i) the external surfaces of the snout bones are poorly ornamented with low relief ridges mostly orientated anteroposterly; (ii) sub-globidont dentition; (iii) apicobasally aligned enamel ridges immediately adjacent to the apical anastomosed region that are closely packed on both the labial and lingual tooth surfaces; and (iv) uniform inter-alveolar spaces in the maxillae that are proportionally narrow relative to alveoli. None of these characteristics are found in the French or German specimens (table 2). They indicate that the Swiss and Portuguese material belongs to the same diagnostic taxon.

**Table 2.** Comparison of dental morphologies and alveolar counts among *Machimosaurus* specimens.

species	*M. buffetauti*			*M. mosae*		*M. hugii*	
reference	[17]	[10]	[3]	[62]	[4]	[6]	[63]
premaxillary alveoli count	3	3	3	3	3	?	?
maxillary alveoli count	>18	22^*a*^	21^*a*^	probably 17 or 18^*b*^	probably 18 or 19^*d*^	at least 18	?
maxillary alveoli anterior to palatines	?	?16–17	?	?7^*c*^	14?	at least 12	?
inter-alveolar spaces between the maxillary alveoli	variable in size, some large	variable in size, some large	?	variable in size, some large	variable in size, some large	posterior–mid inter-alveolar spaces uniform in size	posterior–mid inter-alveolar spaces uniform in size. Anterior spaces larger but still uniform in size
dentary alveoli count	?	21/22	approx. 24/25^*a*^	probably 18^*b*^	19	?	>15
dentary alveoli anterior to the splenial	?	13	?	?	11	?	>8
dentary alveoli adjacent to mandibular symphysis	?	19–20	?	?	15–16	?	>13
diastema between fourth and fifth dentary alveoli	?	yes	?	?	yes	?	?
inter-alveolar spaces between the dentary alveoli	variable in size, some large	variable in size, some large	?	?	variable in size, some large	?	mostly uniform in size, and small
sub-globidont dentition	no	no	no	no	no	yes	yes
apicobasal ridges immediately adjacent to the apical anastomosed region: closely packed on both the labial and lingual surfaces	no	no	no	no	no	yes	yes

^*a*^Estimate [3].

^*b*^We estimate there to be 17 or 18 maxillary alveoli, and most likely a similar number of dentary alveoli. This is higher than the 16 maxillary and dentary alveoli estimate of Sauvage & Liénard [62].

^*c*^The seven pre-palatine maxillary count is based on the figure in Sauvage & Liénard [62]. However, this count assumes that the reconstruction is accurate, which it may not be. This is due to the unusual shape of the anterior process of the palatines and the breaks in the specimen.

^*d*^Hua [4] estimated there to be 17 maxillary alveoli. As the posterior maxillary alveoli are small in *Machimosaurus* [10], a maxillary tooth count of 18 or 19 is likely.

## 6. *Steneosaurus bouchardi* skull of von Huene [63]

**Figure 41.**
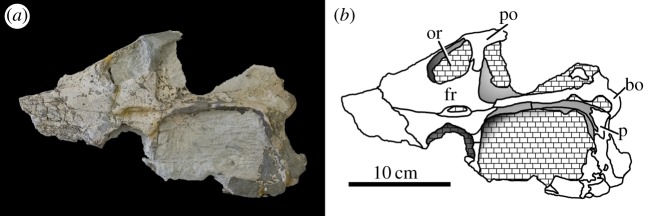
*Machimosaurus hugii*, NMS 7029, referred specimen. Incomplete skull (orbital and temporal region) in dorsal view, (*a*) photograph and (*b*) line drawing. bo, basioccipital; fr, frontal; or, orbit; p, parietal; po, postorbital.

**Figure 42.**
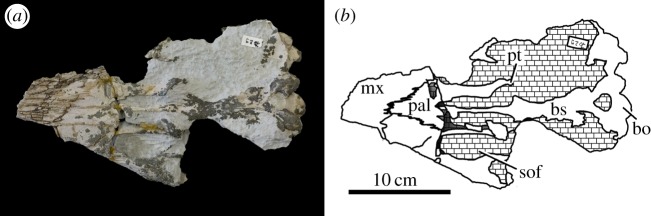
*Machimosaurus hugii*, NMS 7029, referred specimen. Incomplete skull (orbital and temporal region) in ventral view, (*a*) photograph and (*b*) line drawing. bo, basioccipital; bs, basisphenoid; max, maxilla; pal, palatine; pt, pterygoid; sof, suborbital fenestra.

**Figure 43.**
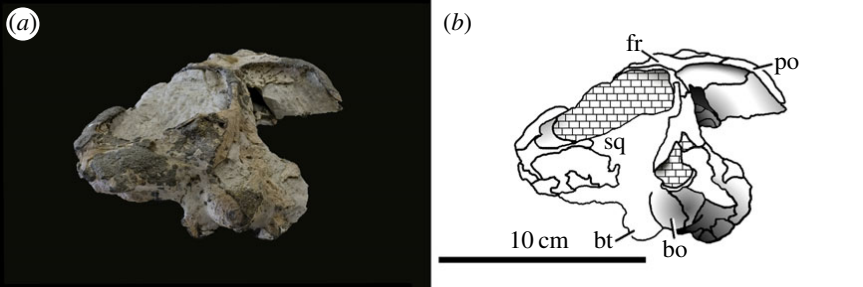
*Machimosaurus hugii*, NMS 7029, referred specimen. Incomplete skull (orbital and temporal region) in occipital/posterior view, (*a*) photograph and (*b*) line drawing. bo, basioccipital; bt, basioccipital tuberosities; fr, frontal; po, postorbital; sq, squamosal.

von Huene [63] also described an incomplete skull and mandible from Solothurn and referred them to *Steneosaurus bouchardi*, an Upper Kimmeridgian species of longirostrine teleosaurid. The skull (NMS 7049; figures 41–43) lacks most of the snout, right supratemporal arch, both quadrates and the palatal surface is poorly preserved. Its specimen labels show that ‘Zangerl, Chicago’ referred this skull to *M. hugii* in 1947.

The Solothurn skull (NMS 7049) shares three autapomorphies with the braincase of the Leiria skull (MG-8730-2): (i) paroccipital processes are greatly enlarged, both elongated mediolaterally and with lateral ends that are expanded; (ii) basioccipital tuberosities (basal tubera) are very large in size and are sub-rectangular in shape when seen in occipital view; and (iii) the inter-basioccipital tubera notch is a large inverse ‘U’-shape when seen in occipital view. Moreover, like the other Swiss specimens and the Portuguese specimen, the external surfaces of the skull bones are poorly ornamented. These features support the referral of NMS 7049 to the same taxon as the Swiss and Portuguese material described above. As we show below that this taxon is *M*. *hugii*, we therefore conclude that Zangerl's referral of NMS 7049 to *M*. *hugii* is correct.

## 7. Systematic palaeontology

Crocodylomorpha Hay, 1930 [67]

Thalattosuchia Fraas, 1901 [68]

Teleosauridae Geoffroy, 1831 [69]

*Machimosaurus* von Meyer, 1837 [31] emend. von Meyer, 1838 [66].

### 7.1 Type species

*Machimosaurus hugii* von Meyer, 1837 [31] emend. von Meyer, 1838 [66].

### 7.2 Referred species

*Machimosaurus buffetauti* sp. nov., *M. mosae* Sauvage & Liénard, 1879 [62] and *Machimosaurus nowackianus* (von Huene, 1938 [19]) comb. nov.

### 7.3 Etymology

‘Pugnacious lizard’. *Machimo* is derived from the Greek word *machimoi* ($\mu \overset{´}{\alpha }\chi \iota \mu o\iota$), meaning pugnacious (Krebs [6] translated it into German as streitbar). In the Hellenic world, *machimoi* was used to describe non-Greek armies, especially native Egyptian troops during the Ptolemaic Dynasties.*Saurus* is the Latinized form of *sauros* (*σαυρoς*), the Ancient Greek for lizard.

### 7.4 Geological range

Middle Oxfordian to Lower Tithonian. (As noted above, we cannot confirm the presence of *Machimosaurus* in deposits younger than the Lower Tithonian.)

### 7.5 Geographical range

Africa (Ethiopia) and Europe (England, France, Germany, Portugal, Spain and Switzerland).

### 7.6 Generic diagnosis

Teleosaurid crocodylomorphs with the following unique combination of characters (autapomorphic characters among teleosaurids are indicated by an asterisk *): large body size (basicranial length typically 90–110 cm, but can exceed 140 cm)*; three alveoli per premaxilla (shared with *Peipehsuchus teleorhinus*); the first premaxillary alveoli are orientated strongly anteroventrally*; 18–22 alveoli per maxilla*; 19–25 alveoli per dentary*; vertically orientated, interlocking dentition, with pronounced reception pits at the premaxillary, maxillary and dentary inter-alveolar spaces*; conical teeth with blunt/rounded apices (shared with ‘*S*.’ *obtusidens*); tooth enamel ornamentation varies along the crown, in the basal region enamel ornamentation is composed of numerous apicobasally aligned ridges of high relief, which become an anastomosed pattern in the apical region (shared with ‘*S*.’ *obtusidens*); presence of carinae is variable, in anterior teeth they can be prominent but in shorter blunter teeth carinae are either very poorly developed or absent*; ratio of crown apicobasal height to basal transverse width can be as low as 1.5 in the posterior teeth; rostrum is broad and mesorostrine, constituting less than 60% of basicranial length*; antorbital fenestrae are absent (possibly shared with *Steneosaurus heberti*); supratemporal fossae are parallelogram in shape (shared with ‘*S*.’ *obtusidens*); ratio of maximum supratemporal fossa length to basicranial length is greater than 27%*; three sacral vertebrae (possibly shared with ‘*S*.’ *obtusidens*)*; medial quadrate hemicondyle is considerably smaller than the lateral hemicondyle*; exoccipital is excluded from the occipital condyle (composed solely of the basioccipital) (shared with *S. heberti*); axis neural spine is tall in lateral view, terminating in a transverse plane that is notably dorsal to the plane of the pre- and postzygapophyses*; axis neural spine posteriorly expanded when seen in lateral view, with the posterior margin terminating approximately in the same frontal plane as the posterior margin of the postzygapophyses*.

### 7.7 *Machimosaurus* sp.

#### 7.7.1 Specimens

MG23—partial maxilla (Malhão, Algarve, south Portugal; Oxfordian) [18].

ML1208—isolated tooth (Middle Oxfordian of Cesaredas, central west Portugal).

Musée de la Princerie (Verdun, France) 2007.0.14—incomplete lower jaw and isolated tooth crowns (Upper Oxfordian, *Perisphinctes* variocostatus subzone of the *P. cautisnigrae* N–W European ammonite zone. From Haudainville, near Verdun, Département de la Meuse, Lorraine, France) (figure 2) [12].

NHMUK PV R36793—isolated tooth (Upper Oxfordian of Villerville, Département du Calvados, Basse-Normandie, France; Calcaire gréseux d'Hennequeville Formation) [17].

#### 7.7.2 Geological range

Middle—Upper Oxfordian.

#### 7.7.3 Geographical range

Europe (France and Portugal).

*Note.* The Oxfordian *Machimosaurus* material is taxonomically indeterminate.

### 7.8 *Machimosaurus hugii* von Meyer, 1837 [31] emend von Meyer, 1838 [66]

**Table:** 

v	1824	Dent obtuse d'un crocodile du Jura, peut-être d'une espèce différence de la précédente [crocodile de Caen]—Cuvier [64, planche 6 fig. 7]
v*	*1837*	*Madrimosaurus hugii* sp. nov.—von Meyer [31, p. 560]
v	*1838*	*Machimosaurus hugii* von Meyer—von Meyer [66, p. 415]
v	*1888*	*Machimosaurus hughi* von Meyer—Lydekker [70, p. 103] (sic)
v	1897–97	*Machimosaurus hugii* von Meyer—Sauvage [18, p. 27, plate 3 figs 9–10 and plate 5 figs 6–7]
v	1925	*Machimosaurus hugii* von Meyer—von Huene [63, pp. 584–588, plate 25 all figures]
v	1925	*Steneosaurus bouchardi* Sauvage—von Huene [63, p. 589, plate 26 fig. 1*a*–*c*]
v	1943	*Machimosaurus hugii* von Meyer—Teixeira [22, p. 109, fig. 1]
v	1967	*Machimosaurus hugii* von Meyer—Krebs [6, pp. 46–58, figs 1–4]
v	1968	*Machimosaurus hugii* von Meyer—Krebs [7, pp. 21–53, figs 1–18]
v	1973	*Machimosaurus hugii* von Meyer—Steel [71, pp. 25, 30, fig. 14 (8) (*partim*)]
v	*2008*	*Machimosaurus hugii* von Meyer—Pierce *et al*. [9, p. 1085 (*partim*)]
v	2010	cf. *Machimosaurus* sp.—Ruiz-Omeñaca *et al*. [48, pp. 81–81, fig. 1*d*]

#### 7.8.1 Lectotype

NMS 8342: isolated tooth crown (figure 28). Previously catalogued as specimen number 96 [6].

#### 7.8.2 ‘Holotype’/syntypes

von Meyer [31,66] never designated a holotype for *M. hugii*. When establishing *M. hugii*, he referred isolated tooth crowns from Solothurn, Switzerland and Kahlenberg, Germany to the species (i.e. a type series or syntypes). Note that Steel [71] mistook Kahlenberg, Hannover as being Kahlenberg in Austria. There are, to our knowledge, no *Machimosaurus* specimens known from Austria.

Therefore, Pierce *et al*. [9] and Martin & Vincent [10] were incorrect in referring to a holotype for this taxon. They appear to have considered the lectotype as being the holotype, as the same specimen number is given (the old 96 number which Krebs [6] used). Also, Pierce *et al*. [9] stated that the ‘holotype’ is from the Palaeontologische Sammlung im Museum der Stadt Solothurn, the former name for the Naturmuseum Solothurn (again, the old name which Krebs [6] used). Martin & Vincent [10], however, listed the ‘holotype’ as being from the Staatliche Naturhistorische Sammlungen Dresden, which is the former name for the Senckenberg Naturhistorische Sammlungen Dresden. No reason is stated why they believed that the ‘holotype’ was moved to a different museum in a different country. Regardless, we can confirm that the lectotype tooth is still in Solothurn.

#### 7.8.3 ‘Neotype’

More confusingly, Martin & Vincent [10, p. 192] claimed that Krebs [6,7] made the Portuguese specimen (MG-8730-1, MG-8730-2) the neotype of *M. hugii*. However, Krebs [6]: (i) never refers to a neotype and (ii) clearly referred to the Swiss tooth (NMS 8342, then catalogued as specimen number 96) as the ‘lectotypus’ of *M. hugii*. Moreover, this is the earliest mention of a lectotype we can find for *M. hugii*, and Krebs [6] may have designated it acting as first reviser. Moreover, in Krebs' later paper, he still refers to the isolated tooth crown as being the lectotype [7, p. 35 and figured on p. 37]. Other than in Martin & Vincent [10], we can find no reference to a *M. hugii* ‘neotype’.

#### 7.8.4 Lectotype locality

Kreuzen Quarry at St. Verena, near Solothurn, Canton Solothurn, Switzerland [6]; 47° N, 7° E.

#### 7.8.5 Lectotype horizon

‘Rätschenbank’ der Schildkrötenschichten [6] (= Solothurn Turtle Limestone, Reuchenette Formation). Uppermost Kimmeridgian, Upper Jurassic.

#### 7.8.6 Etymology

‘Hugi's pugnacious lizard’. Named in honour of Franz Joseph Hugi (1791–1855), the Swiss geologist and naturalist.

#### 7.8.7 Referred specimens

MG-8730-1, MG-8730-2 and unnumbered elements—incomplete skull and postcranial elements (Lower or Upper Kimmeridgian of Guimarota near Leiria, Portugal [6,7]; figures 4–10).

ML491, ML495, ML959 and ML1955—isolated teeth (Upper Kimmeridgian of Porto das Barcas, Lourinhã, Portugal; Praia Azul Member of the Lourinhã Formation; figures 33*a* and 35).

ML647—isolated tooth crown (Upper Kimmeridgian of Peralta, Lourinhã, Portugal; Praia Azul Member of the Lourinhã Formation; figure 34).

ML657 and ML658—isolated teeth (Upper Kimmeridgian of Zimbral, Lourinhã, Portugal; Praia Azul Member of the Lourinhã Formation; figure 33*b*–*d*).

ML647, ML733 and ML902—isolated teeth (Upper Kimmeridgian of beach near Lourinhã, Portugal; Praia Azul Member of the Lourinhã Formation).

MUJA-1008 and MUJA-1922—isolated teeth (Kimmeridgian of Playa de La Griega, Colunga, Asturias, Spain; Tereñes Formation; figure 11*c*,*d*) [25].

MUJA-1298—isolated tooth crown (Kimmeridgian of La Escalera, Villaviciosa, Asturias, Spain; Lastres Formation; figure 11*a*–*d*) [25].

MCNV-CC-4—isolated tooth crown (Tithonian of Cantera Carcalín near Buñol, Valencia Province, Spain [48]).

From the Kimmeridgian of Solothurn, Switzerland: NHMUK PV OR33239, NHMUK PV OR43638, NHMUK PV R5, NHMUK PV R232—isolated tooth crowns (figure 29). NMS 7012—incomplete snout (figures 37 and 38). NMS 7015—incomplete snout (maxilla-palatine fragment; figure 39). NMS 7021—incomplete mandible (figure 40). NMS 7029—temporal and orbital region of a skull (figures 41–43).

#### 7.8.8 Geological range

(Lower Kimmeridgian?) Upper Kimmeridgian—Lower Tithonian.

#### 7.8.9 Geographical range

Europe (Portugal, Spain and Switzerland).

#### 7.8.10 Species diagnosis

Teleosaurid crocodylomorph within the genus *Machimosaurus* with the following unique combination of characters (autapomorphic characters are indicated by an asterisk *): the external surfaces of the skull bones are poorly ornamented, in particular those of the rostrum and around the orbits*; sub-globidont dentition*; apicobasally aligned enamel ridges immediately adjacent to the apical anastomosed region that are closely packed on both the labial and lingual tooth surfaces*; moderate post-symphyseal dentary tooth count (three to four pairs); inter-alveolar spaces between the maxillary and dentary alveoli are very small (closely packed alveoli)*; the premaxilla is notably wide at the level of the external nares, much wider than the width of the anterior end of the maxilla*; orbits are sub-rectangular in shape*; paroccipital processes are greatly enlarged, both elongated mediolaterally and with lateral ends that are expanded*; basioccipital apophysis has a ‘U-shaped’ cross section (teleosaurid symplesiomorphy); basioccipital tuberosities (basal tubera) are very large in size and are a sub-rectangular shape in occipital view*; the inter-basioccipital tubera notch is a large inverse ‘U’-shape when seen in occipital view*; dorsal osteoderm ornamentation is composed of small-to-large irregularly shaped pits arranged in a random manner, that are well separated from one another (somewhat similar to *Steneosaurus leedsi*).

### 7.9 *Machimosaurus buffetauti* sp. nov.

**Table:** 

v	*1873*	*Steneosaurus burgensis* nomen nudum—Jarrin ([72], pp. 103–104)
v	*1876*	*Steneosaurus burgensis* nomen nudum—Jarrin ([73], pp. 94–96)
v	1905	*Steneosaurus burgensis Chanti* nomen nudum—Chanel ([74], pp. 17–39), figs 1–3
v	1982	*Machimosaurus hugii* von Meyer—Buffetaut ([2], pp. 19–22), plate 1 figs A–D
v	1982	*Machimosaurus hugii* von Meyer—Buffetaut ([3], pp. 17–24), plate 1
v	2004	*Machimosaurus hugii* von Meyer—Karl & Tichy [8], figs 1, 2
v	2006	*Machimosaurus hugii* von Meyer—Karl *et al*. ([32], pp. 67–69), fig. 8
v	2008	*Machimosaurus hugii* von Meyer—Lepage *et al*. ([17], pp. 116–118), figs 1–7
v	*2008*	*Machimosaurus hugii* von Meyer—Pierce *et al*. ([9], p. 1085) (*partim*)
v	2013	*Machimosaurus hugii* von Meyer—Martin & Vincent ([10], pp. 179–196), figs 1–9

#### 7.9.1 Holotype

SMNS 91415: complete skull and mandible, with partial postcranial skeleton (figures 22–27).

#### 7.9.2 Holotype locality

Am Hörnle Quarry, Neuffen, Baden-Württemberg, Germany [10].

#### 7.9.3 Holotype horizon

Lacunosamergel Formation, *A. hypselocyclum* Sub-Mediterranean ammonite Zone (Weißer Jura gamma 2), Lower Kimmeridgian, Upper Jurassic [10].

#### 7.9.4 Etymology

‘Buffetaut's pugnacious lizard’. Named in honour of Eric Buffetaut (b. 1950), whose research has greatly elucidated thalattosuchian and crocodylomorph evolution.

#### 7.9.5 Referred specimens

MPV V.1600.Bo and V.1601.Bo—anterior half of rostrum (premaxilla, maxilla and dentary) in occlusion and a maxilla-nasal fragment (Calcaires Coquilliers Formation; *P*. *baylei* Sub-Boreal ammonite Zone, lowermost Kimmeridgian of Cricqueboeuf, Normandy, Northern France; figures 12–14) [2,17].

DFMMh FV 330, DFMMh FV 541: isolated tooth crowns (Langenberg Formation; Langenberg near Oker, Lower Saxony, Germany; Kimmeridgian; figure 21*c*–*h*) [8,32].

Musée de Brou (Bourg-en-Bresse, France), specimen number unknown—a complete skull and mandible in articulation (Calcaires à ptérocères Formation, Lower Kimmeridgian; Montmerle, Bourg-en-Bresse, département de l'Ain, France [3]).

#### 7.9.6 Geological range

Lower Kimmeridgian.

#### 7.9.7 Geographical range

Europe (France and Germany). An isolated tooth from Smallmouth Sands, England (NHMUK PV R1774; figure 21*a*,*b*) is possibly referable to this taxon, as are isolated teeth from Czarnogłowy, Poland (GPIT/RE/328, GPIT/RE/9280 and GPIT/RE/9281; figures 31 and 32).

#### 7.9.8 Species diagnosis

Teleosaurid crocodylomorph within the genus *Machimosaurus* with the following unique combination of characters (autapomorphic characters are indicated by an asterisk *): 21–22 alveoli per maxilla (approx. 16–17 of which are anterior to the palatines); 24/25 alveoli per dentary (19–20 of which are adjacent to the mandibular symphysis); low post-symphyseal dentary tooth count (two pairs)*; inter-alveolar spaces between the maxillary and dentary alveoli are variable in size (thalattosuchian symplesiomorphy); orbits are sub-circular in shape (transverse and anteroposterior axes are sub-equal; the *Steneosaurus brevior* holotype also has circular orbits)*; the quadrates have a single large circular depression on the dorsal surface near the hemicondyles*; basioccipital apophysis has a ‘U-shaped’ cross section (teleosaurid symplesiomorphy); the inter-basioccipital tubera notch is a wide and gentle inverse semicircle when seen in occipital view (teleosaurid symplesiomorphy); basioccipital tuberosities (basal tubera) are reduced in size when seen in occipital view (apomorphy shared with *M. mosae*); axis neural arch dorsal margin is strongly concave when seen in lateral view*; axis postzygapophyses terminate significantly posterior to posterior surface of the centrum view (somewhat similar to that seen in *S. durobrivensis*); coracoid glenoid process (process near the glenoid fossa that projects posterodorsally) is elongate, extending considerably from the head of the coracoid, and is a sub-isosceles triangle in shape when seen in lateral view*; coracoid postglenoid process anterior margin is very slightly concave and terminates approximately in the same frontal plane as the glenoid*; coracoid postglenoid process posterior margin is strongly concave and terminates approximately in the same frontal plane as the posterior end of the glenoid process*; dorsal osteoderm ornamentation is composed of small-to-large irregularly shaped pits arranged in a random manner, that are well separated from one another (somewhat similar to *S. leedsi*).

*Steneosaurus burgensis*. The names *S. burgensis* and *S. burgensis chanti* have been applied to the *Machimosaurus* skull from Ain, France [3,72–74]. These specific and sub-specific names are however *nomina nuda*. Both Jarrin [72] and Chanel [74] stated that the Ain skull was sent to Caen for preparation and study by Eugène Eudes-Deslongchamps, who proposed the name *S. burgensis* for the specimen, in consultation with the Société d'émulation de l'Ain. Neither Jarrin [72,73] nor Chanel [74] established the name under Article 12 of the International Commission on Zoological Nomenclature (ICZN) Code as: they did not describe the specimen, nor did they provide a definition of the species; they simply reported that the name was proposed by Eudes-Deslongchamps. Unfortunately, Eudes-Deslongchamps never published his description of the Ain skull [3,74]. The specimen was not formally described until 1982, and then it was referred to *M. hugii* [3]. The sub-specific epithet *chanti* was apparently established by those who did not fully understand zoological nomenclature [3], as it was added to the Ain skull's specimen plaque solely to honour the discoverer [3,74].

Our decision to establish a new taxon based on SMNS 91415, and not formally establish *S. burgensis* for the Ain skull, was for several reasons: (i) the Ain skull is partially reconstructed, and it is unclear how much is plaster and how much is real bone [3]; (ii) the cranium and lower jaw of the Ain skull are in articulation, meaning that the palatal and dorsal mandibular morphologies cannot be seen [3,74]; (iii) the German skull SMNS 91415 has the cranium and lower jaw disarticulated, allowing these morphologies to be observed [10]; and (iv) SMNS 91415 has associated postcranial material, greatly aiding in comparisons with other *Machimosaurus* taxa, in particular *M. mosae*.

### 7.10 *Machimosaurus mosae* Sauvage & Liénard, 1879 [62]

**Table:** 

	1876	*Teleosaurusmosae* sp. nov.—Liénard (manuscript name)
v*	1879	*Machimosaurus mosae* comb. nov.—Sauvage & Liénard [62, pp. 1–31, plates 1–4]
v	*1973*	*Machimosaurus hugii* von Meyer—Steel [71, p. 25 (*partim*)]
v	1993	*Machimosaurus mosae* Sauvage & Liénard—Hua *et al*. [21, pp. 851–856, texte-fig. 1]
v	1999	*Machimosaurus mosae* Sauvage & Liénard—Hua [4, pp. 141–170, figs 1 and 2, plates 1–6]
v	*2009*	*Machimosaurus mosae* Sauvage & Liénard—Pierce *et al*. [9, p. 1085]

#### 7.10.1 Holotype

Much of the skeleton: incomplete skull, mandible, 22 vertebrae, part of the pelvis, numerous ribs, limb bones and 22 osteoderms. The specimen disappeared during the First World War [20] and is presumed to have been destroyed.

#### 7.10.2 Holotype locality and horizon

Issoncourt, near Verdun, Département de la Meuse, Lorraine, France. The specimen most likely comes from the *A. autissiodorensis* Sub-Boreal ammonite zone, ‘Marnes supérieures de la Meuse’ [4].

#### 7.10.3 Neotype

An almost complete skeleton: nearly complete skull, mandible, half of the cervical vertebrae, all the dorsal and sacral vertebrae, approximately a third of the caudal vertebrae, two chevrons, cervical and dorsal ribs, left scapula, right coracoid, right fibula, both pubes, both ilia, left ischium, right femur, left tibia, and dorsal and ventral osteoderms [21] (figures 15–20).

#### 7.10.4 Neotype locality

A beach near Ambleteuse, Boulonnais, Département du Pas-de-Calais, Nord Pas-de-Calais, France.

#### 7.10.5 Neotype horizon

Argiles de Châtillon Formation [4,21]. From either the *A. autissiodorensis* Sub-Boreal ammonite zone, uppermost Kimmeridgian, or the *G. gigas*/*P. elegans* Sub-Boreal ammonite zone, lowermost Tithonian.

#### 7.10.6 Neotype note

The neotype was originally catalogued as BHN2R 1100. While the BHN2R closed in 2003, the neotype was removed from the museum prior to this. It is assumed that the neotype is now in a private collection, but this cannot be confirmed. A cast of the neotype is on display in RBINS. It was purchased from Eldonia Paléontologie, and it is unclear how much is based on the original specimen (P. Godefroit 2014, personal communication). The original cast was made by the University of Paris 6—Université Pierre-et-Marie-Curie (E. Buffetaut 2014, personal communication), and we are unsure how Eldonia Paléontologie obtained a copy.

#### 7.10.7 Etymology

‘Pugnacious lizard of the Meuse’. Named after the French river Meuse, near which the holotype was discovered.

#### 7.10.8 Previously referred specimen

Lydekker [70] referred an incomplete skull and mandible (NHMUKPV R1089) from Upper Kimmeridge Clay Formation (Early Tithonian) of England to *M. mosae*. This specimen, however, was recently shown to belong to the metriorhynchid *P. manselii* [41].

#### 7.10.9 Geological range

Uppermost Kimmeridgian and/or lowermost Tithonian.

#### 7.10.10 Geographical range

Europe (northeastern France).

#### 7.10.11 Species diagnosis

Teleosaurid crocodylomorph within the genus *Machimosaurus* with the following unique combination of characters (autapomorphic characters are indicated by an asterisk *): 17–18 alveoli per maxilla (approx. 14 of which are anterior to the palatines); 19 alveoli per dentary (15–16 of which are adjacent to the mandibular symphysis); moderate post-symphyseal dentary tooth count (three to four pairs); inter-alveolar spaces between the maxillary and dentary alveoli are variable in size (thalattosuchian symplesiomorphy); orbits are transverse ellipsoids in shape (anteroposterior axis is 79.7% the length of the transverse axis)*; prearticular is absent*; basioccipital apophysis has a ‘V-shaped’ cross section*; the inter-basioccipital tubera notch is a wide and gentle inverse semicircle when seen in occipital view (teleosaurid symplesiomorphy); basioccipital tuberosities (basal tubera) are reduced in size when seen in occipital view (apomorphy shared with *M. buffetauti*); axis neural arch dorsal margin is subtly concave when seen in lateral view (somewhat similar to that seen in *S. leedsi*); axis postzygapophyses terminate only slightly posterior to posterior surface of the centrum*; coracoid glenoid process is very short, not extending far from the head of the coracoid and is a right-angled triangle in shape when seen in lateral view*; coracoid postglenoid process anterior margin is strongly concave and terminates in a frontal plane anterior to the glenoid*; coracoid postglenoid process posterior margin is strongly concave distally but shifts to being somewhat convex proximally and terminates in a frontal plane posterior to the posterior end of the glenoid process*; dorsal osteoderm ornamentation is composed of numerous small, irregularly shaped pits arranged in an anastomosed pattern, these pits can fuse and become elongate grooves that radiate from the keel (similar to ‘*S*.’ *obtusidens*)*; ventral osteoderms have a longitudinal keel*.

#### 7.10.12 Validity of *Machimosaurus mosae*

Martin & Vincent [10, p. 193] claimed that *M. mosae* was an invalid name, based upon Articles 8 and 9 of the Code of the ICZN. Their contention was based on the 1876 manuscript of Liénard entitled ‘Le *Teleosaurus Mosae*, fossile des marnes kimméridgiennes de la Meuse’ [62, p. 7], which was the first to use the name *T. Mosae*. Sauvage & Liénard [62, p. 7] stated that Liénard's description remained in manuscript form and was sent to the Ministry of Public Education at the end of 1876, ‘ont été indiqués par lui dans un travail resté manuscrit et adressé à la fin de l'année 1876 au Ministère de l'Instruction publique’.

Martin & Vincent [10] are correct that Sauvage & Liénard [62, p. 11] used the following headings:

Deuxieme Partie.—Description du Machimosaurus mosæ, *F. Liénard* sp. (1).

However, this is key to why *T. mosae* is an available name under the ICZN Code. Immediately below those headings is a detailed species diagnosis, followed by an eight-page description of the skeleton, a long discussion on the affinities of *Machimosaurus*, and four plates with line drawings of the specimen. As such, along with the paper being published in a scientific journal, this description clearly fulfils the criteria set out in Articles 8, 11 and 12 of the ICZN Code.

Article 11.6 of the ICZN Code, publication as synonymy, states: ‘A name which when first published in an available work was treated as a junior synonym of a name then used as valid is not thereby made available’. Article 11.6.1 states: ‘However, if such a name published as a junior synonym had been treated before 1961 as an available name and either adopted as the name of a taxon or treated as a senior homonym, it is made available thereby but dates from its first publication as a synonym’. Clearly, Sauvage & Liénard [62] treated *T. mosae* as an available name, and they adopted its specific name for their validly described taxon. Consequently, under Article 11.6.1 *M. mosae* is an available name.

However, the authorship is Sauvage & Liénard, 1879 not Liénard, 1876. This is due to Article 50.7 of the ICZN Code, which states: ‘If a scientific name (taken, for example, from a label or manuscript) was first published in the synonymy of an available name and became available before 1961 through the provisions of Article 11.6, its author is the person who published it as a synonym, even if some other originator is cited, and is not the person who subsequently adopted it as a valid name.’ Therefore, *contra* Martin & Vincent [10], *M. mosae* is not invalid, and the nominal authors of the specific name are indeed Sauvage & Liénard [62].

#### 7.10.13 Tooth taxa of Sauvage [11]

Sauvage [11] listed three *Machimosaurus* species living in the Late Kimmeridgian–Early Tithonian of Northern France (around Boulogne-sur-Mer): *M. hugii*, *M. interruptus* and *M. ferox*. The latter two were established by Sauvage [11] for isolated tooth crowns from the area. Krebs [8, p. 48] stated that Sauvage invoked insignificant differences between the crowns when establishing his species and that Sauvage himself later withdrew the name *M. ferox*. Owing to the geological age and location of these isolated teeth, it is possible they are referable to *M. mosae*. If so, *M. interruptus* would have priority. As the holotype of *M. interruptus* cannot be found, and the neotype of *M. mosae* is currently unavailable for study, this possibility cannot be explored.

### 7.11 *Machimosaurus nowackianus* (von Huene, 1938 [18]) comb. nov.

**Table:** 

v	1938	cf. *Simolestes nowackianus* sp. nov. —von Huene [19, pp. 370–376, figs 1–4]
v	1960	*Simolestes nowackianus* von Huene—Tarlo [75, pp. 173, 183, fig. 3*c*]
v	1996	*Machimosaurus* sp. —Bardet & Hua [20, pp. 65–71, figs 1–2]

#### 7.11.1 Holotype

GPIT Orig. Huene 1938 figs 1–4: anterior region of the dentary (figure 3). Note that the specimen currently cannot be located in GPIT.

#### 7.11.2 Holotype locality

Near Feyambiro, east of Harrar, Harrar Province, Ethiopia [19].

#### 7.11.3 Holoype horizon

It is not clear what formation the specimen was found in [19,20]. It was described as being found in sandy clays, 8 m above a crystalline basement.

#### 7.11.4 Etymology

‘Nowack's pugnacious lizard’. Named in honour of the holotype's discoverer, Mr Nowack.

#### 7.11.5 Geological range

Oxfordian or Kimmeridgian.

#### 7.11.6 Geographical range

Africa (Ethiopia).

#### 7.11.7 Species diagnosis

Teleosaurid crocodylomorph within the genus *Machimosaurus* with the following unique combination of characters (autapomorphic characters are indicated by an asterisk *): the anterior dentary inter-alveolar spaces are reduced, being notably smaller in size than in *M. buffetauti* and *M. mosae*, in particular the D1–D2 and D2–D3 inter-alveolar spaces which are both less than half the length of the D2 alveoli*; the interdentary distance between the D3 and D4 alveolar couplets is notably smaller than in *M. buffetatuti* and *M. mosae*, such that the transverse width of the left and right D4 alveoli are as large as, or greater than, the immediately adjacent flat dentary region*.

*Note.* While the two anterior dentary characteristics readily differentiate *M. nowackianus* from both *M. buffetauti* and *M. mosae*, no known *M. hugii* specimen preserves the anterior region of the dentary.

## 8. Discussion

### 8.1 Ontogeny

The differences in tooth count, symphyseal length and length of the supratemporal fossae enable easy identification for two of the four *Machimosaurus* species (*M. buffetauti* and *M. mosae*; table 1). Interestingly, there are three skulls of *M. buffetauti* and *M. mosae* between 93 and 100 cm in basicranial length. All of these have a snout (=preorbital) length to basicranial length ratio of approximately 58%, even though there is variation in maxillary tooth count, dentary tooth count, dentary symphyseal tooth count and mandibular symphysis length. This shows that proportional snout length does not vary between these two species (even though these dental and mandibular characteristics do vary). This indicates that the differences between these two species are not due to ontogeny, because it would be unusual for juveniles and adults of the same taxon to have nearly identical skull lengths and nearly identically proportioned snouts. Moreover, when we look beyond tooth counts and biometric ratios and examine cranial, mandibular and postcranial morphologies, we find numerous characteristics that differentiate these two species (see diagnoses and tables 2 and 3).

**Table 3.** Comparison of craniomandibular and postcranial morphologies among *Machimosaurus* specimens.

species	*M. buffetauti*		*M. mosae*		*M. hugii*	
reference	[10]	[3]	[62]	[4]	[6]	[63]
orbit shape	circular	circular	transverse ellipsoid	transverse ellipsoid	?	sub-rectangular
quadrate dorsal surfaces have a large circular depression	yes	?	?	no	no	?
paroccipital processes	similar in size and shape to other teleosaurids	similar in size and shape to other teleosaurids	similar in size and shape to other teleosaurids	similar in size and shape to other teleosaurids	mediolaterally elongate and the lateral ends are greatly expanded	mediolaterally elongate and the lateral ends are greatly expanded
basioccipital condyle apophysis cross section	‘U’-shaped	‘U’-shaped	?	‘V’-shaped	‘U’-shaped	?
basioccipital tuberosity size	small	?	?	small	very large	very large
inter-tuber notch (basioccipital tuberosity) shape in occipital view	semicircular, mediolaterally wide	?	?	semicircular, mediolaterally wide	inverse ‘U’-shape, mediolaterally narrow	inverse ‘U’-shape, mediolaterally narrow
prearticular	present	?	?	absent	?	?
axis neural arch dorsal margin in lateral view	strongly concave	?	?	subtly concave	?	?
coracoid glenoid process, shape in lateral view	sub-isosceles triangle	?	?	right-angled triangle	?	?
coracoid postglenoid process, anterior margin shape	very slightly concave, in the same frontal plane as the glenoid	?	?	strongly concave, in a frontal plane that is anterior to the glenoid	?	?
coracoid postglenoid process, posterior margin shape	strongly concave, in the same frontal plane as the posterior end of the glenoid process	?	?	strongly concave distally and becomes convex proximally, in a frontal plane posterior to the posterior end of the glenoid process	?	?
dorsal osteoderm ornamentation pattern	small-to-large sub-circular pits that are well separated	?	?	tightly packed, small, sub-circular pits arranged in an anastomosed pattern	small-to-large sub-circular pits that are well separated	?

**Figure 44.**
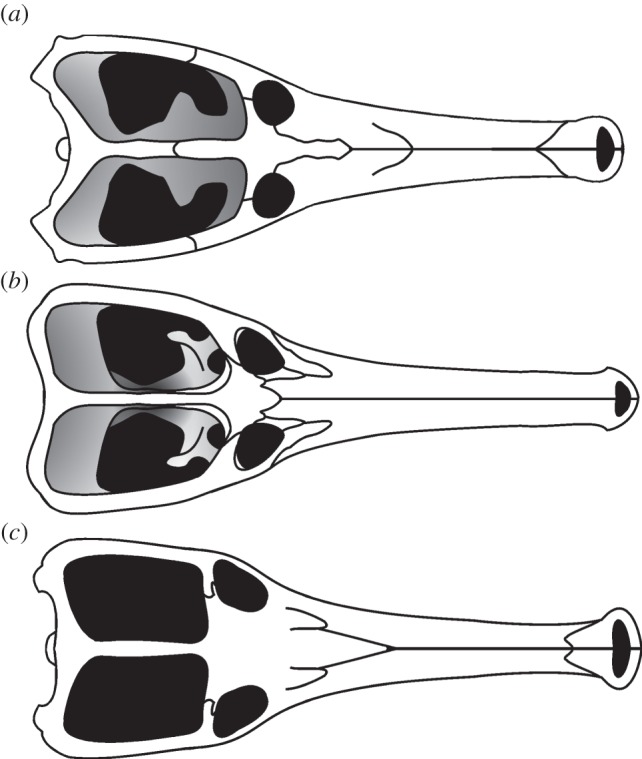
Reconstructions of the skulls of the European *Machimosaurus* species in dorsal view. (*a*) *Machimosaurus mosae* (the neotype), (*b*) *M. buffetauti* (the holotype SMNS 91415) and (*c*) *M. hugii* (based on MG-8730-1, MG-8730-2, NMS 7012 and NMS 7029).

This leaves the type species *M. hugii* as a remaining point of discussion for the European species. Unfortunately, the postcranial characteristics that differentiate *M. buffetauti* and *M. mosae* are unknown in *M. hugii* (table 3), as are the differences in cranial biometrics and tooth counts (tables 1 and 2). *Machimosaurus hugii* does have three braincase apomorphies: (i) paroccipital processes are greatly enlarged, both elongated mediolaterally and with lateral ends that are expanded; (ii) basioccipital tuberosities (basal tubera) are very large in size; and (iii) inter-basioccipital tubera notch is a large inverse ‘U’-shape when seen in occipital view. *Machimosaurus hugii* also has two dental apomorphies: (i) sub-globidont dentition and (ii) apicobasally aligned enamel ridges immediately adjacent to the apical anastomosed region that are closely packed on both the labial and lingual tooth surfaces. These five characteristics readily allow *M. hugii* to be identified, and seem unlikely to be under ontogenetic control (for example, a drastic change between non-globidont and sub-globidont dentition is not known in any other crocodylomorph and would be greatly unexpected). Therefore, it is unlikely that *M. hugii* is the adult version of either *M. buffetauti* or *M. mosae*. As further support of this conclusion, we note that the smaller *M. buffetauti* and *M. mosae* individuals do not show any juvenile characteristics (such as having proportionally large orbits to basicranial length or paired frontals (P. Vignaud 1995, unpublished PhD thesis)). Moreover, there is variation in orbit shape, frontal shape and premaxilla width (figure 44).

### 8.2 Age and locality

**Figure 45.**
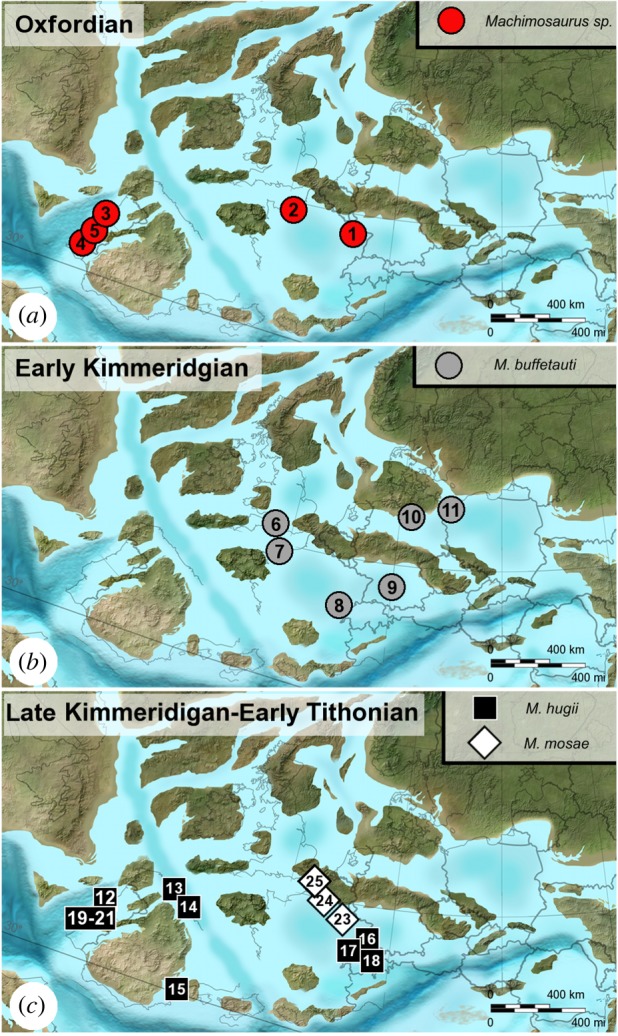
Map of *Machimosaurus* specimens from across Europe, (*a*) in the Oxfordian, (*b*) the Early Kimmeridgian and (*c*) during the Late Kimmeridgian–Early Tithonian. Localities listed below with an asterisk (*) denote where we are not certain that specimens from that locality can be referred to the listed species. *Machimosaurus* sp.: (1) Haudainville near Verdun, Lorraine, France; (2) Villerville, Normandy, France; (3) Cesaredas, central west Portugal; (4) Malhão, Algarve, Portugal; (5) Cesareda, Portugal. *Machimosaurus buffetauti*: (6) Smallmouth Sands, Dorset, England*; (7) Cricqueboeuf, Normandy, France; (8) Montmerle, Bourg-en-Bresse, France; (9) Neuffen, Baden-Württemberg, Germany; (10) Langenberg near Oker, Germany; (11) Czarnogłowy, West Pomerania, Poland. *Machimosaurus hugii*: (12) Guimarota, Leiria, Portugal; (13) La Escalera, Villaviciosa, Asturias, Spain; (14) Playa de La Griega, Colunga, Asturias, Spain; (15) Cantera Carcalín near Buñol, Valencia Province, Spain; (16) Solothurn, Canton Solothurn, Switzerland; (17) Moutier, Canton Bern, Switzerland*; (18) Porrentruy, Canton Jura, Switzerland*; (19–22) Santa-Cruz, Porto das Barcas, Peralta and Zimbral, Lourinhã area, Portugal. *Machimosaurus mosae*: (23) Issoncourt, near Verdun, Lorraine, France; (24) Mont-Lambert near Boulogne-sur-Mer, France*; (25) beach near Ambleteuse, Boulonnais, France. Palaeomaps were modified version of high-resolution versions kindly provided by Ron Blakey (http://cpgeosystems.com/).

The four *Machimosaurus* species were not spatio-temporally contemporaneous. All of the specimens we refer to *M. buffetauti* are from the Lower Kimmeridgian of France and Germany (and possibly also England and Poland, although we cannot confirm this at present). *Machimosaurus mosae* is known from the final Sub-Boreal ammonite zone of the Kimmeridgian and/or the first Sub-Boreal ammonite zone of the Tithonian, and only known from northeastern France. As such, *M. buffetauti* and *M. mosae* were separated in time by 3–5 million years (figure 45).

The lectotype of *M. hugii* is known from the Solothurn turtle limestone, a member of the Reuchenette Formation (Switzerland), which was deposited during the final Sub-Mediterranean ammonite zone of the Kimmeridgian. This suggests that *M. hugii* and *M. mosae*were probably contemporaneous in age, but lived in different European provinces, with *M. hugii* in the Sub-Mediterranean realm and *M. mosae* in the Sub-Boreal realm (figure 45).

The other *M. hugii* specimens are known from Portugal and Spain. As noted above, the exact age of the Leiria specimen is unknown, with it being either Early or Late Kimmeridgian in age. The Lourinhã specimens are close to the Kimmeridgian–Tithonian boundary. Isolated Spanish teeth are Kimmeridgian and Early Tithonian in age [25,48]. All the Iberian specimens have the same dental morphologies as the uppermost Kimmeridgian Swiss specimens (apicobasally aligned enamel ridges immediately adjacent to the apical anastomosed region that are closely packed on both the labial and lingual tooth surfaces [6,48]).

*Machimosaurus nowackianus* is the only known non-European taxon, and thus was widely separated from the three European taxa. It is currently difficult to assess whether it may have been temporally separated from the European taxa as well, as the age of the Ethiopian deposits is only coarsely constrained to the Oxfordian–Kimmeridgian. In summary, all four *Machimosaurus* species were either separated by several million years in time or by hundreds or thousands of kilometres.

### 8.3 Body size

The neotype of *M. mosae* gives the first definitive evidence of body length in this genus. The skeleton was approximately 6 m in length and had a basicranial length of 96.5 cm [21]. This therefore allows us to make an estimate of total body length for any *Machimosaurus* specimen with a basicranial length (assuming skull-to-body length scaling remains consistent). These estimates reveal something quite interesting about three of the four species (table 4). The largest known *M. buffetauti* skull is from Ain, France [3]. At 100 cm long, it gives a body length estimate of 6.22 m. The holotype of *M. buffetauti* has a basicranial length of 93.5 cm [10]. This results in a slightly smaller body length of 5.81 m.

**Table 4.** Estimated total body lengths for *Machimosaurus* specimens. Estimate based on the ratio of the basicranial length (96.5 cm) to total body length (approx. 600 cm) of the *M. mosae* neotype [4,21].

species	references	basicranial length (cm)	body length estimate (m)
*M. hugii*	[6,7]	149	9.26
*M. mosae*	[62]	130	8.08
*M. buffetauti*	[3]	100	6.22
	[10]	93.5	5.81

While the neotype of *M. mosae* is approximately 6 m in body length, the lost holotype was far larger. It had an estimated basicranial length of 130 cm [62]. This results in a body length estimate of 8.08 m, making it substantially larger than the older *M. buffetauti*. The type species *M. hugii* was larger still. The incomplete Leira skull was estimated to have a basicranial length of 149 cm [6]. This gives a body length estimate of 9.26 m. As such, Krebs' [6,7] estimate of the Leira specimen exceeding 9 m long in length was reasonable.

**Figure 46.**
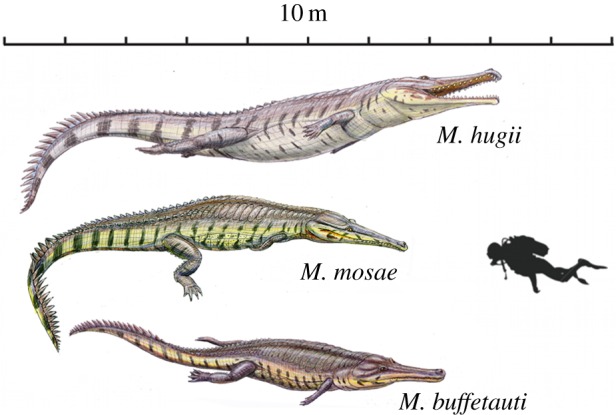
Life reconstructions showing the maximum body lengths of the three *Machimosaurus* species present in the Kimmeridgian–Tithonian of Europe. The human diver is 1.8 m in height. The life reconstructions were made by Dmitry Bogdanov.

Therefore, there were three European species of *Machimosaurus*, which appear to have differed in total body length (figure 46). The Lower Kimmeridgian species *M. buffetauti* is both the geologically oldest and smallest of the three *Machimosaurus* species, with a body length in the range of 5.8–6.2 m. The uppermost Kimmeridgian–lowermost Tithonian species *M. mosae* was larger, with a body length in the range of approximately 6–8 m. The Upper Kimmeridgian–Lower Tithonian species *M. hugii* was the largest of all these species, with a body length exceeding 9 m. Unfortunately, *M. nowackianus* is only known from an anterior dentary, so its body length cannot be reliably estimated. However, this hypothesis requires further testing as new specimens are discovered, because the sample size used here is small (although it includes all known complete skulls).

Therefore, at over 9 m in length, *M. hugii* is the largest known crocodylomorph of the Triassic and Jurassic Periods, and until the Cretaceous it was the largest crocodylomorph that had ever existed in Europe.

### 8.4 Hypothetical lifestyles

Two very different lifestyles have been hypothesized for this genus. Krebs [6,7] presented evidence that the Leiria *M. hugii* specimen was adapted for living in open seas, whereas Hua [4] put forward evidence that the *M. mosae* neotype was adapted for living in coastal, high-energy environments. These two hypothetical lifestyles are very different and contradictory. If Martin & Vincent [10] were correct and there is only one *Machimosaurus* taxon, this would present a major anomaly. However, as we have already shown, *Machimosaurus* taxa differ in craniomandibular, dental and postcranial morphologies. They also differed in geological age, locality and total body length. As such, there is overwhelming evidence that there were three non-sympatric *Machimosaurus* species in Europe during the Kimmeridgian, and there is no reason why these distinct species could not have had distinct lifestyles.

Krebs [6,7] postulated a convincing argument that the Leiria *M. hugii* specimen was well suited to an open sea lifestyle: based on the vertebral zygapophyseal articulations, *M. hugii* would have been well suited to swimming by lateral undulations of the tail, perhaps using the limbs for steering and balancing, and was a fast swimmer. Also, the cervicocranial depressor musculature would have been well developed, as their attachment sites on the skull were enlarged (the basioccipital tubera and the paroccipital processes), which would have greatly assisted *Machimosaurus* in diving [7]. Krebs' [6,7] hypothesis that *M. hugii* was well suited to an open sea lifestyle helps explain this species’ initially odd geographical distribution: the northern Tethyian and eastern Proto-Atlantic margins (Switzerland, Spain and Portugal).

An additional line of evidence that *M. hugii* was better suited to a more aquatic/pelagic lifestyle comes from dermal bone and osteoderm ornamentation. The ornamentation of dermal bones and osteoderm pits in extant crocodylians are known to be vascularized [76–78]. Infrared thermal imaging of basking broad-snouted caimans (*Caiman latirostris*) demonstrates heat exchange between osteoderms and the environment. Moreover, at low temperatures (16°C) the dorsal surface of the snout is one of the warmest regions of the body, whereas at higher temperatures (25°C) it no longer is [78]. Furthermore, the distribution of dermal ornamentation on the skull of Pseudosuchia in general is consistent with the hypothesis that the most exposed (dorsal) parts of the skull are the most ornamented (M. Fau, M. Laurin and V. de Buffrénil 2014, personal communication). Therefore, vascularized dermal bone/osteoderm dorsal surfaces, heat exchange and blood flow alternation appear to have a role in thermoregulatory terrestrial basking behaviours [76–78]. However, as noted above, skull and osteoderm ornamentation of *M. hugii* was reduced (also see [7, fig. 17]) suggesting it was less vascularized than other *Machimosaurus* species. This supports current research on skull ornamentation in crocodylomorphs, which found an inverse relationship between regional dermal ornamentation levels and aquatic specialization [79].

This relationship certainly exists within the thalattosuchian clade Metriorhynchoidea, in which basal members have highly ornamented crania [80], while within the pelagic clade Metriorhynchidae there is repeated evolution of a ‘smooth’ skull [81]. Metriorhynchoids have a more extreme shift in thermoregulatory behaviour, as basal metriorhynchoids have osteoderms and retain external mandibular fenestrae (which enables the musculus intramandibularis of extant crocodylians to fix the jaws in a gaping position during mouth-gaping basking behaviour), whereas metriorhynchids lack osteoderms and the external mandibular fenestrae [80].

Hua [4] postulated a convincing lifestyle for the *M. mosae* neotype. The robust ribs, thick and keeled ventral osteoderms, thick gastralia and three sacral vertebrae would have helped *M. mosae* remain in place in a high-energy/turbulent environment [4]. The small paroccipital processes and basioccipital tubera show that this species was not well suited for diving [4,21]. Moreover, the high number of tightly packed dorsal osteoderm pits and the well-ornamented skull suggest that *M. mosae* had a highly vascularized dorsal surface, well suited for terrestrial basking behaviours.

No hypothetical lifestyle has been postulated for *M. buffetauti*. However, this taxon is similar to *M. mosae* (tables 1–3). However, comparing the figures of the ventral osteoderms and ribs in Hua [4] to those in Martin & Vincent [10] reveals that *M. mosae* was a more robust taxon than *M. buffetauti*. Interestingly, the dermal bone and osteoderm ornamentation of *M. buffetauti* is more similar to *M. hugii* than *M. mosae*, suggesting that this Lower Kimmeridgian species was perhaps intermediate between the two Upper Kimmeridgian extremes. As *M. nowackianus* is only known from an anterior dentary, no hypothetical lifestyle can be postulated.

## 9. Conclusion

We here review and clarify the systematics of *Machimosaurus*, one of the most distinctive teleosaurid crocodylomorphs from the Jurassic. We show that *M. mosae* is indeed an available name. We also show that the type specimen (the lectotype) of *M. hugii* is an isolated tooth crown from Switzerland, which is still curated at Solothurn, and that the Portuguese specimen was not considered to be the neotype of this species. We also established *M*. *buffetauti* sp. nov. for the Lower Kimmeridgian specimens from France and Germany, based on a previously described specimen. Most importantly, we demonstrate that there were three species of *Machimosaurus* in the Kimmeridgian (Upper Jurassic) of Europe, and another taxon in Ethiopia. This conclusion is not solely based on tooth counts and cranial biometric ratios, unlike recent revisions of the genus. Our revision uses these characteristics, but expands upon them to include comparative anatomy (craniomandibular, dental and postcranial), body size, hypothetical lifestyle, geological age and geographical range. This holistic approach readily identifies three non-sympatric European species and reveals that potentially contemporaneous taxa were adapted for very different ecosystems. What is surprising, however, is that much of the reasoning we outline here has been long known and thoroughly described, but had not been synthesized.

One new aspect that helps differentiate *Machimosaurus* species in this paper is postcranial morphology. Prior to description of the *M. buffetauti* holotype by Martin & Vincent [10], the only postcranial skeleton that was well described and figured was the *M. mosae* neotype [4]. We have attempted to begin to rectify the long neglected study of teleosaurid postcranial morphology here, as we show for the first time that the postcranial skeletons of *M. buffetauti* and *M. mosae* are distinct (table 3). We recommend that future studies on thalattosuchians do not solely focus on snout length, tooth counts and biometric ratios of skull measurements, but thoroughly investigate craniomandibular and postcranial morphologies.

As *Machimosaurus* specimens are becoming increasingly abundant, one potentially interesting hypothesis could be investigated with future discoveries. Was *Machimosaurus* in the Kimmeridgian–Lower Tithonian equivalent to the Pliocene–Holocene Asian–Australasian and American subclades of *Crocodylus*? The extant Asian–Australasian *Crocodylus* subclade has one large-bodied taxon well suited to traversing marine barriers (*C. porosus*) and five geographically limited taxa across its range (*C. johnsoni*, *C. mindorensis*, *C. novaeguineae*, *C. palustris* and *C. siamensis*), while the extant American *Crocodylus* subclade has one large-bodied taxon well suited to traversing marine barriers (*C. acutus*) and three geographically limited taxa across its range (*C. intermedius*, *C. moreletii* and *C. rhombifer*) [82]. In *Machimosaurus*, there is one large-bodied taxon well suited to traversing marine barriers (*M. hugii*), with geographically limited but temporally distinct taxa living in Europe and Ethiopia. Could there be more geographically limited *Machimosaurus* taxa along the margins of Tethys and/or palaeo-islands? It is a potentially interesting hypothesis that will require future study.

Recent phylogenetic analyses place *Machimosaurus* and ‘*S*.’ *obtusidens* as sister taxa, forming a clade of durophagous/generalist teleosaurids that lived during the Callovian–Tithonian. Their large size, robust craniomandibular and dental morphologies, and broad dietary range suggest ‘*S*.’ *obtusidens* and *Machimosaurus* were an important component of Jurassic shallow marine/brackish ecosystems. We anticipate that future discoveries, especially from outside of Europe, will further elucidate the evolution of this remarkable clade of marine crocodylomorphs.
